# Native musk and synthetic musk ketone strongly induced the growth repression and the apoptosis of cancer cells

**DOI:** 10.1186/s12906-016-1493-2

**Published:** 2016-12-08

**Authors:** Ling Xu, Yi Cao

**Affiliations:** Laboratory of Molecular and Experimental Pathology, Kunming Institute of Zoology, Chinese Academy of Sciences, 32 Jiaochang Donglu, Kunming, Yunnan 650223 China

**Keywords:** Traditional Chinese medicine, Musk, Musk ketone, Cancer, Proliferation, Apoptosis, Differentially expressed gene, IL-24, DDIT3

## Abstract

**Background:**

Musk is widely used in clinical practice for its anti-cancer properties. Here, we treated various types of cancer using musk to determine which cancers are sensitive to musk treatment. We also compared effects of native musk and synthetic musk ketone in cancer cells. Furthermore, we investigated mechanisms underlying effects of musk.

**Methods:**

Twenty two cancer cell lines were treated with musk. Cell proliferation and apoptosis analyses were carried out. Native musk and synthetic musk ketone were analyzed by gas chromatograph-mass spectrometer (GC-MS) assay. Differentially expressed genes were determined by microarray and quantitative real–time polymerase chain reaction.

**Results:**

Native musk strongly induced the growth repression and the apoptosis in the majority of cancer cell lines in a dose-dependent manner, but distinct types of cancer showed significantly different reactions. Cancer cells which originated from epithelial cells showed higher sensitivity for musk treatment. By contrast, leukaemia and lymphoma cells were not sensitive. GC-MS analysis demonstrated that native musk contains more than 30 contents in which musk ketone is a major component; synthetic musk ketone was consistent with natural musk ketone, and the used sample of synthetic musk ketone contained only sole component. Similar to native musk, synthetic musk ketone induced the growth repression and the apoptosis of cancer cells. Additionally, numerous genes were differentially expressed in lung cancer cells after native musk treatment. These differentially expressed genes were involved in many signalling pathways. Among these pathways, apoptosis-related pathways included interleukin family, tumor necrosis factor family, and MAPK signalling pathway. Native musk and synthetic musk ketone can up-regulate IL-24 (interleukin family) and DDIT3 (MAPK signalling pathway) in lung cancer cells.

**Conclusions:**

This research provided strong evidence that native musk and synthetic musk ketone can induce the growth repression and the apoptosis of cancer cells. However, the selection of sensitive cancer patient for individualized treatment is a key step in clinical application. Synthetic musk ketone can substitute for native musk to treat cancer patients. Musk might induce the growth repression and the apoptosis of lung cancer cells through up-regulating IL-24 and DDIT3 expressions.

**Electronic supplementary material:**

The online version of this article (doi:10.1186/s12906-016-1493-2) contains supplementary material, which is available to authorized users.

## Background

Cancer is one of the vital causes of death worldwide. Anti-cancer drugs play important roles in cancer treatment. Although increasing numbers of anti-cancer drugs have been applied in clinical practice, most cancer patients presented poor prognosis because of lack of effective treatment. Therefore, novel anti-cancer drugs should be developed. Traditional Chinese medicine (TCM) has been used to treat gas heavy diseases (now regarded as malignant tumors by modern medicine) for thousands of year. From TCM, several effective anti-cancer drugs had been discovered and developed, such as arsenic trioxide (As2O3) for treatment of acute promyelocytic leukaemia [1, 2] and camptothecin for solid tumours [3]. And some new cancer treatments were studied [4, 5]. TCM possesses advantages in specific aspects at a certain stage of cancer treatment [6]. Musk (Shexiang, *Moschus*), as a common and valuable TCM, is widely applied to treat various diseases. Clinically, musk is used for anti-bacterial, anti-inflammatory, immunity-enhancing, and treatments of gas heavy diseases in clinical practice. As recorded in several ancient TCR books such as “Shen Nong Ben Cao Jing (The Herbal Classic of the Divine Plowman)” and “Compendium of Material Medicine”, musk exerts a therapeutic effect for gas heavy diseases. In the 1970s, musk had been studied for the treatment of gastrointestinal cancer [7]. In the 1990s, a TCM named ‘compound TIANXIAN capsule’ which contained musk as the main ingredient, was used to treat gastrointestinal tumors and had shown a certain effect [8]. In a previous study, musk was buried within the abdomen after radical gastrectomy for stomach cancer, and this treatment significantly prolonged the survival time of patients [9]. Recently, several reports demonstrated that Xihuang pill which contains musk, revealed anti-breast cancer effects [10]. Moreover, *Toona sinensis* and *Moschus* decoction induced cell cycle arrest in HeLa cells [11].

Although musk treatment was effective for tumor patients, some cancer patients did not respond to musk treatment in clinical applications. Thus, cancer sensitivity should be determined in order to enhance the effectiveness of musk therapy. In the present study, we treated various cancer cell lines with musk to determine which cancers were sensitive to musk treatment. The results may be helpful to select appropriate treatments for patients and to guide clinical therapy.

Native musk is obtained from the capsule gland of male musk deer and its source is very limited. Thus, the use of synthetic compound instead of native musk provides a great significance for clinical application. Native musk primarily contains musk ketone, nitrogen-containing compounds, cholesterol, fatty acids, and inorganic salts. These materials have anti-bacterial, anti-inflammatory, immunity-enhancing, and anti-tumor effects [12]. Musk ketone, a major component of native musk, had been used for cancer treatment. A previous study found that synthetic musk ketone could significantly inhibit the growth of breast cancer cells in a nude mouse model [13]. In the current study, we compared responses of cancer cells to native musk and synthetic musk ketone in vitro.

Although musk treatment suppressed the tumor growth in clinical application, and experimental studies confirmed that musk could inhibit cancer cell proliferation and trigger apoptosis [7–9, 14]. However, mechanisms underlying these effects remain unknown. In this study, we also investigated signalling pathways associated with the growth inhibition and the apoptosis in lung cancer cells treated by musk.

## Methods

### Native musk and synthetic musk ketone

The native musk sample was obtained from the gland capsule of a dead musk deer. This musk deer died of natural causes. The dead musk deer was provided by Li-Jiang City, Yunnan Province, China. The musk deer belongs to Moschus berezovskii. The study was approved by the Ethics Committee for Animal Experimentation, Kunming Institute of Zoology, Chinese Academy of Sciences. The native musk sample (0.076 g) was added to 1 ml of ethanol and the mixture was shaken for 1 hour (h). The supernatant was filtered through a 0.22 μm filter and stored at 4 °C. Gas chromatograph-mass spectrometer-computer (GC-MS) analysis confirmed that main ingredients of native musk were extracted. The sample of synthetic musk ketone (the purity: 98%) was purchased from Chengdu Preferred Biotechnology. Co. Ltd (CAS: 541-91-3, Lot No.13709; Chengdu, China), and this sample was dissolved in ethanol, filtered through a 0.22 mm filter and stored at 4 °C. The chemical structure of synthetic musk ketone was showed in Additional file 1.

### Cell lines and cell culture

Up to 22 human cancer cell lines were used in this study. These cell lines included 11 types of cancer such as lung squamous cell carcinoma, lung adenocarcinoma, lung large cell carcinoma, lung small cell carcinoma, mammary carcinoma, esophageal carcinoma, gastric carcinoma, colorectal carcinoma, hepatocellular carcinoma, acute myelogenous leukemia, and B cell lymphoma. These cell lines were cultured with RPMI 1640 or DMEM medium (GIBCO Invitrogen, Grand Island, NY, USA) containing 10% foetal bovine serum and maintained in a humidified incubator with 5% CO_2_ at 37 °C. XLA-07 and XL-JT were provided by Dr LJ Ma [15]. Detailed information about the cell lines is presented in Additional file 2.

### Cell proliferation assay

Cultured cells were seeded in 96-well plates at a density of 1 × 10^4^ cells per well. Native musk and synthetic musk ketone (four replicates in each group) were added at varying concentrations. After 24 h of treatment, 20 μl of MTS solution [3-(4,5-dimethylthiazol-2-yl)-5-(3-carboxymethoxyphenyl)-2-(4-sulfophenyl)-2H-tetrazolium, inner salt; Promega Corporation, Madison, WI, USA] was added to each well, and the plates were incubated for 4 h at 37 °C. Absorbance at 490 nm was measured using a spectrophotometer. The same amount of solvent (ethanol) was used as the control.

### Flow cytometry (FCM) analysis

Apoptotic cells were determined by FCM analysis with the Fluorescein Isothiocyanate (FITC)-labeled Annexin V Kit (BD Pharmingen, San Diego, CA, USA) in accordance with the manufacture’ instruction. Treated cells were washed twice with phosphate buffer saline (PBS) and adjusted at a density of 5 × 10^5^ cells/100 μl. Cell suspensions were added to each tube; afterward, the cells were stained with annexin V-FITC and propidium iodide and then analyzed under FCM (BD Biosciences, San Jose, CA, USA). Collected data for 10,000 cells and WinMDL software were used for the analysis of FCM data files.

### GC-MS analysis

The native musk sample was extracted with ethanol as described above. The supernatant was filtered and determined by GC-MS (Agilent Technologies, Palo Alto, CA, USA; HP6890GC/5973MS) as previous described [16]. Similarly, 1 μl of the musk ketone sample was dissolved in 100 μl of ethanol and then determined by GC-MS. Qualitative analysis was performed with the Wiley7n.l standard library.

### mRNA expression profiling

Harvested cells were washed twice with cold PBS, and total RNA was isolated using Trizol reagent (TaKaRa, Tokyo, Japan). Determination of mRNA profiling was performed in Eplc-32M1 and XL-JT cells with or without native musk treatment by using Agilent Human Gene Expression array (CapitalBio Technology, Beijing, China; http://www.capitalbio.com). Differentially expressed genes were subjected to Gene Ontology (GO) as well as Kyoto Encyclopedia of Genes and Genomes (KEGG) pathway analyses using Molecule Annotation System (MAS) 3.0 (http://bioinfo.capitalbio.com/mas3).

### Quantitative real–time polymerase chain reaction (qRT-PCR)

For mRNA expression assay, total RNA was isolated from cells by using TRlzol reagent (TaKaRa). Synthesis of cDNA was carried out by M-MLV Reverse Transcriptase (Promega) with random primer and amplified with specific primers on the StepOne Realtime PCR System (Applied Biosystems, Foster City, CA, USA). QRT-PCR analysis was performed using miScript SYBR Green PCR kit (QIAGEN, Hilden, Germany) in accordance with the manufacturer’s instructions. The PCR reaction process was first incubated at 95 °C for 3 minutes (min), followed by 40 cycles of thermal cycling at 95 °C for 15 seconds (s) and 60 °C for 30 s. The mRNA levels of the target genes were demonstrated using ΔC_T_ (ΔC_T_ = C_T, Target_-C_T, actin_; C_T, Target,_ the average threshold cycle number of the target gene; C_T, actin,_ the average threshold cycle number of the β-actin). The primer sequences were described as follows. TNFRSF25: 5′-CCCAGAACACACCTACTCTGC-3′(R), 5′-AGAGATACTGACTGT-GGGACC-3′(F); IL-24: 5′-TCCAACTGTTTGAATGCTCTCC-3′(R), 5′-CTTTGTTCT-CATCGTGTCACAAC-3′(F); DDIT3: 5′-CTGCTTGAGCCGTTCATTCTC-3′(R), 5′-GGAAACAGAGTGGTCATTCCC-3′(F).

### Statistical analysis

Statistical significance was calculated using the Student’s *t*-test. SPSS 17.0 software package (Chicago, IL, USA) was used for all statistical analyses. The level of statistical significance was set at 0.05 for all the tests.

## Results

### Native musk treatment inhibited the proliferation of cancer cells and induced the apoptosis

**Fig. 1:**
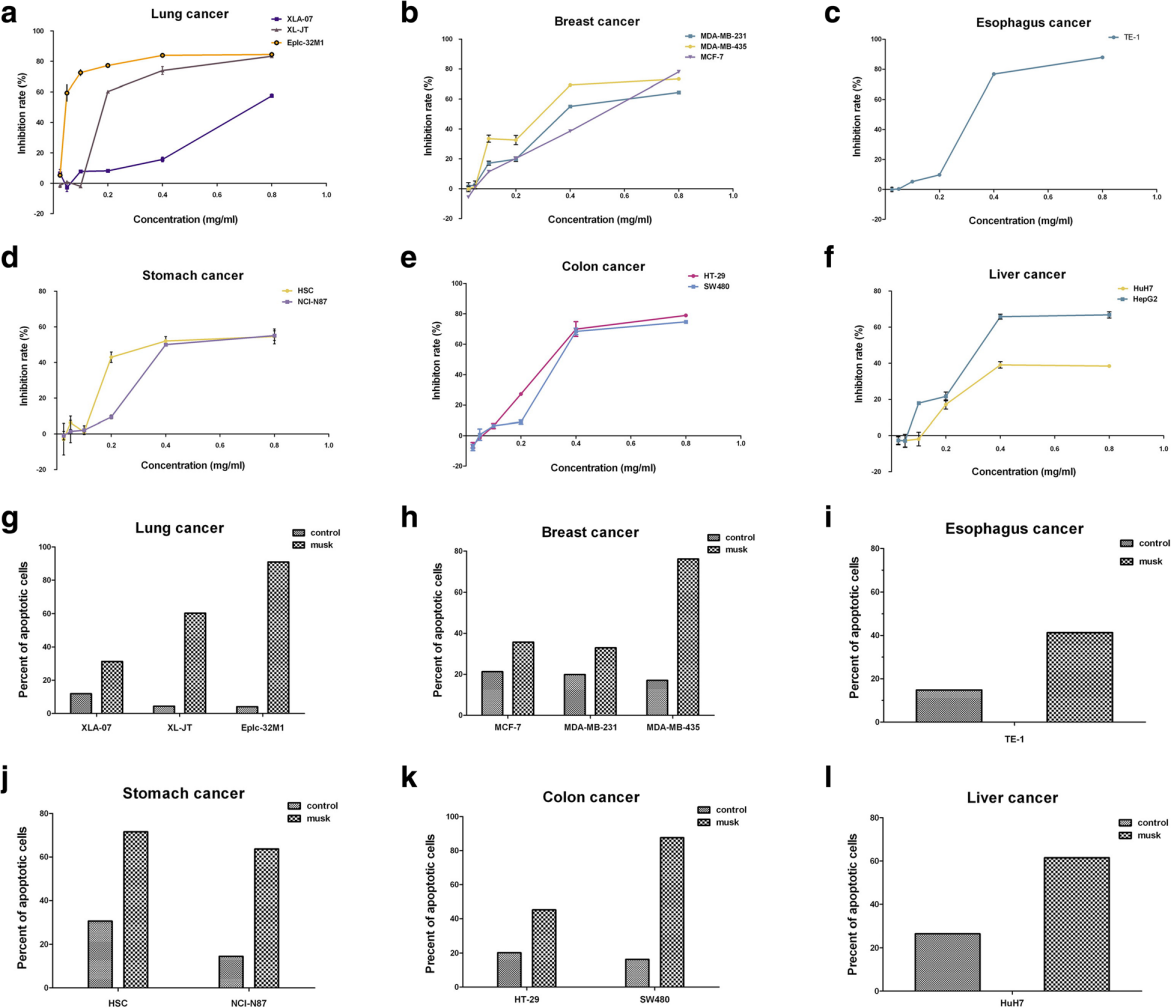
Native musk induced the growth repression and the apoptosis in cancer cell lines. **a**-**f** Native musk inhibited the cellular proliferation in the six carcinoma cell lines, as shown by MTS assay. **g**–**l** Native musk induced the apoptosis in the six carcinoma cell lines, as shown by FCM assay. Student’s t-test, *p* < 0.05

Up to the 22 cancer cell lines were used in this study. Cultured cells were exposed to various concentrations (0.025, 0.05, 0.1, 0.2, 0.4, and 0.8 mg/ml) of native musk for 24 h. The data indicated that musk treatment induced the growth repression in the 17 cancer cell lines in a dose-dependent manner, but did not show any effect in the 5 other cancer cell lines. The results are summarized in Table 1. In particular, native musk strongly inhibited the growth of the three lung cancer cell lines; likewise, the growth of several carcinoma cell lines were strongly suppressed (Fig. 1a to f). Moreover, native musk selectively targeted the 12 cancer cell lines and promoted the apoptosis of cancer cells (Fig. 1g to l).

**Table 1:** Reactivities of various cancer cell lines after treatment with native musk

Cell lines	Reactivities^a^
Lung squamous cell carcinoma	
Eplc-32M1	++++
Lung adenocarcinoma	
GLC-82	++
XLA-07	+++
XL-JT	++++
A549	+++
Lung large cell carcinoma	
NCIH-460	-
801-D	-
Lung small cell carcinoma	
NCIH-446	-
Mammary carcinoma	
MDA-MB-231	+++
MDA-MB-435	+++
MCF-7	++
Esophageal carcinoma	
TE-1	++++
Gastric carcinoma	
HSC	+++
NCI-N87	+++
SGC-7901	+
Colorectal carcinoma	
HT-29	+++
Caco-2	++
SW480	+++
Hepatocellular carcinoma	
Huh7	++
HepG2	+++
Acute myelogenous leukemia	
HL-60	-
B cell lymphoma	
Daudi	-

^a^Reactivities mean inhibition rates of the cellular proliferation after the treatment

The inhibition rates: −, <10%; +, 10–20%; ++, 20–40%; +++, 40–60%; ++++, 60–80%

The concentration of native musk: 0.8 mg/ml

### Synthetic musk ketone inhibited the proliferation of cancer cells and induced the apoptosis

**Fig. 2:**
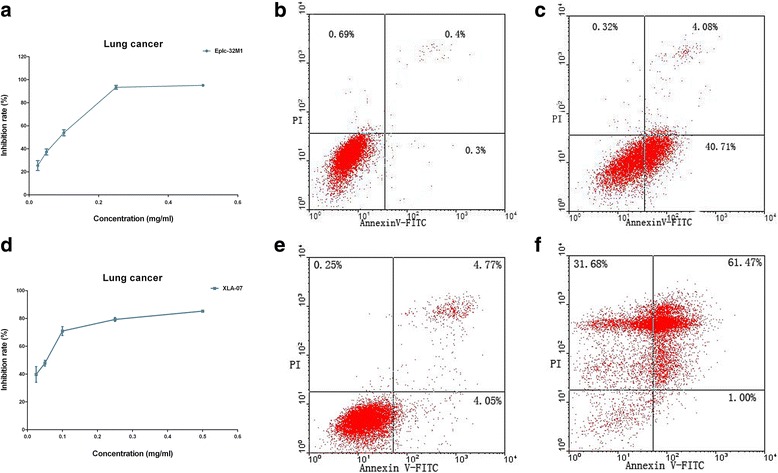
Synthetic musk ketone induced the growth repression and the apoptosis in cancer cell lines. Synthetic musk ketone inhibited the cell proliferation in Eplc-32M1 (**a**; lung squamous cell carcinoma) and XLA-07 (**d**; lung adenocarcinoma). Notably, synthetic musk ketone exerted significant effect at low concentration. Synthetic musk ketone induced the apoptosis in Eplc-32M1 (**b**, **c**) and XLA-07 (**e**, **f**), as shown by FCM assay. **b** and **e** were negative controls. Student’s t-test, *p* < 0.05

In this study, we selected the six cancer cell lines which showed positive response for native musk to analyze effects of synthetic musk ketone. Cultured cells were exposed to various concentrations (0.025, 0.05, 0.1, 0.25, and 0.5 mg/ml) of synthetic musk ketone for 24 h. MTS and FCM assays were performed to examine effects of synthetic musk ketone in the selected cancer cell lines. These results indicated that synthetic musk ketone can also induce the growth repression and the apoptosis in the six cancer cell lines in a dose-dependent manner (Table 2, Fig. 2). Notably, synthetic musk ketone exerted significant effects under low concentration compared with native musk.

**Table 2:** Reactivities of various cancer cell lines after treatment with synthetic musk ketone

Cell lines	Reactivities^a^
Lung squamous cell carcinoma	
Eplc-32M1	++++
Lung adenocarcinoma	
XL-JT	++++
XLA-07	++++
Esophageal carcinoma	
TE-1	++++
Gastric carcinoma	
HSC	++++
Mammary carcinoma	
MDA-MB-435	++++

^a^Reactivities mean inhibition rates of the cellular proliferation after the treatment

The inhibition rates: +, 10–20%; ++, 20–40%; +++, 40–60%; ++++, 60–80%

The concentration of synthetic musk ketone: 0.5 mg/ml

### Native musk and synthetic musk ketone were analyzed and compared by GC-MS assay

**Fig. 3:**
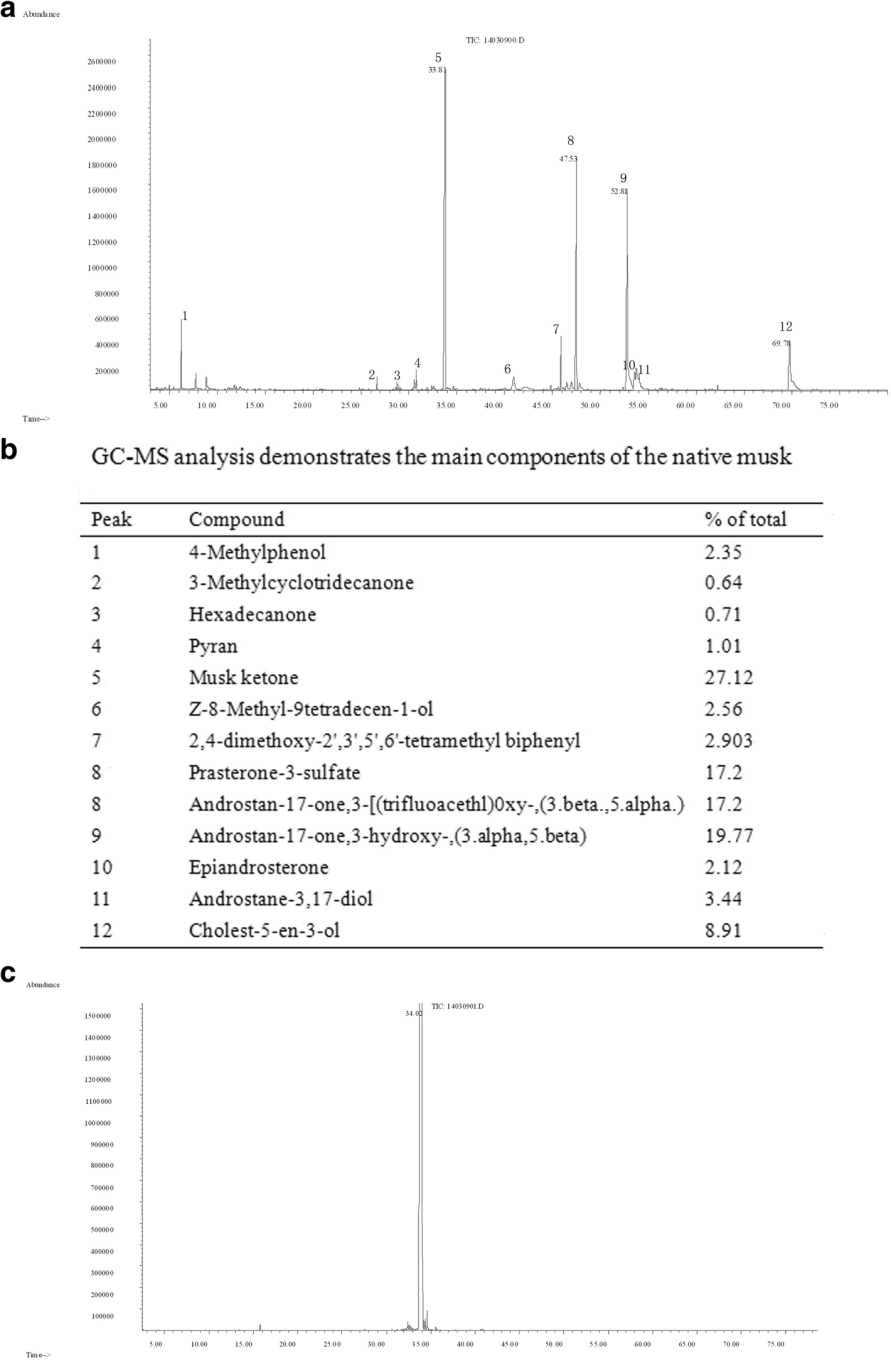
Native musk and synthetic musk ketone were analyzed and compared by GC-MS assay. **a** Native musk contains more than 30 contents, in which the 12 notable peaks are detected. **b** The 12 notable peaks are explained; **c** the sample of synthetic musk ketone used in this study is a single component

Results of GC-MS assay demonstrated that native musk contains more than 30 contents. Up to the 12 notable peaks were selected for further analysis (Fig. 3a). The 12 contents are presented in Fig. 3b. Among the 12 contents, the amount of musk ketone was the highest, reaching 27.12%. Moreover, GC-MS assay revealed that the sample of synthetic musk ketone used in this study contains only one component that is musk ketone (Fig. 3c).

### mRNA expression scan identified differentially expressed genes after native musk treatment

To explore molecular mechanisms associated with musk treatment of lung cancer, we performed microarray analysis to determine mRNA profiles of Eplc-32M1 and XL-JT following native musk treatment. Differentially expressed genes in Eplc-32M1 and XL-JT after the treatment are listed in Additional files 3 and 4, respectively. These differentially expressed genes were grouped into approximate 30 categories. These results indicated that musk has a wide range of biological activities. In this study, we were particularly interested in apoptosis-related signalling pathways because of their potential roles in the growth repression and the apoptosis induced by musk.

### Possible pathways were involved in the apoptosis induced by native musk and synthetic musk ketone

**Fig. 4:**
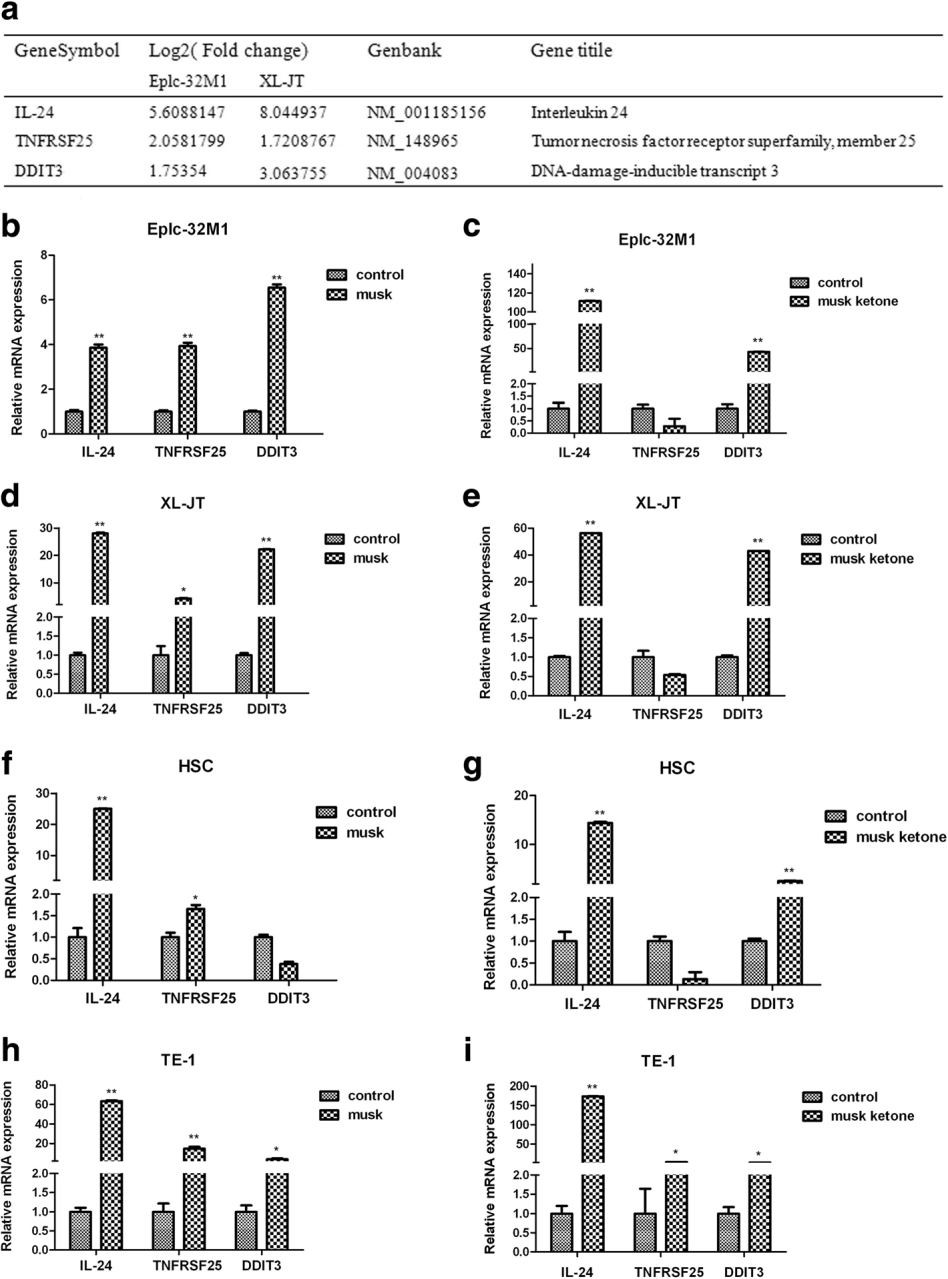
IL-24, TNFRSF25, and DDIT3 were differentially expressed in cultured cancer cells after native musk and synthetic musk ketone treatments. **a** Changes in IL-24, TNFRSF25, and DDIT3 mRNA were determined by microarray analysis in Eplc-32M1 and XL-JT after native musk treatment. **b**–**d** IL-24, TNFRSF25 and DDIT3 mRNA levels were determined by qRT-PCR in Eplc-32M1, XL-JT, HSC, and TE-1 after native musk and synthetic musk ketone treatments; musk, native musk; musk ketone, synthetic musk ketone; control, no treatment. Student’s t-test. **, *p* < 0.01; *, *p* < 0.05

Analyses of differentially expressed genes showed that signalling pathways related with the musk induced-apoptosis included interleukin (IL) family, tumor necrosis factor (TNF) family, MAPK signalling pathway, p53 signalling pathway, and Jak-STAT signalling pathway. The three common genes (IL-24, interleukin family; TNFRSF25, TNF family; DNA-damage-inducible transcript 3 [DDIT3], MAPK signalling pathway) that were differentially expressed in both Eplc-32M1 and XL-JT cells treated with native musk, were selected for further analysis. The mRNA levels of the three genes were verified in Eplc-32M1 and XL-JT cells treated with native musk and synthetic musk ketone via qRT-PCR analyses. The treatment of native musk induced up-regulation of the three genes (IL-24, TNFRSF25, and DDIT3), whereas the treatment of synthetic musk ketone led to up-regulation of the two genes (IL-24 and DDIT3; Fig. 4).

## Discussion

Experiences in TCM for thousand years and researches on modern medicine have demonstrated that musk treatment is effective for tumor therapy [7–9, 14]. However, the therapeutic effect of musk showed significant differences in various cancer patients. The principle of ‘personalized therapy’ or ‘precision medicine’ indicated that the selection of appropriate patient for individualized treatment, particularly for cancer therapy, is of great significance. Distinct types of cancer may show different responses to the same treatment. In the present study, we applied various types of cultured cancer cells to investigate their response to musk treatment. The cancer cells examined in this study belong to lung cancer, esophagus cancer, stomach cancer, colon cancer, liver cancer, breast cancer, leukaemia, and lymphoma. Our results demonstrated that: 1) musk can induce the growth repression and the apoptosis of cancer cells, which provided a clear scientific basis for cancer therapy using musk; 2) the therapeutic effect of musk was dose-dependent. 3) importantly, various cancers showed significantly different responses to musk treatment. For instance, cancer cells which originated from epithelial cells were highly sensitive for musk treatment. By contrast, leukaemia and lymphoma cells were not sensitive. We also emphasized that even for the same type of carcinoma, distinct cell lines displayed significant difference in terms of sensitivity. Thus, we proposed that sensitive patients should be selected for individualized treatment using musk through experimental methods and therapeutic trials.

In this study, we also found that the musk action was dose-dependent, indicating that administration of high doses may enhance the therapeutic effect. However, musk produces certain toxic effects, and administration of high doses may also be harmful to patients. Therefore, the appropriate dose and the method of administration should be considered. Interestingly, in southwestern China, TCM physicians treated severe infections and tumors in lung as well as upper gastrointestinal tract through inhalation and swallowing of small amounts of native musk, respectively (personal communication). This particular mode of administration achieved better treatment effects compared with traditional methods, such as oral and subcutaneous embedding of pills containing native musk or synthetic musk ketone. We suggested that musk may be applied in different ways for treatments of various cancers as follows: swallowing and oral administration for esophagus and stomach cancer, external application for breast cancer, and inhalation for lung cancer.

Native musk is a very rare and precious natural drug. Studying and synthesizing its active ingredients is very important in pharmacology and clinical medicine. Native musk contains more than 30 contents. Among them, musk ketone is a major component, accounting for 27%. Previous research showed that synthetic musk ketone exerted anti-tumor activity [13]. In the current study, we used synthetic musk ketone to treat cultured cancer cells. We found that synthetic musk ketone can also induce the growth repression and the apoptosis of cancer cells, similar to the effect of native musk. Furthermore, GC-MS analysis demonstrated that synthetic musk ketone used in our study was consistent with natural musk ketone contained in native musk. The used sample of synthetic musk ketone only included the sole component. Our study indicated that synthetic musk ketone can substitute for native musk to treat cancer patients, and synthetic musk ketone may be a promising anti-cancer drug. We also noted that native musk contains multiple components. We thought that musk ketone may be synergistic with other components to play an anti-cancer effect during the native musk treatment. However, relevant researches are lacking so far.

Musk possesses a wide range of biological activities and pharmacological effects as well as has been used for anti-bacterial, anti-inflammatory, immunity-enhancing, and anti-cancer treatments [17, 18]. In our study, both native musk and synthetic musk ketone can inhibit the growth of cancer cells and induce the apoptosis. However, mechanisms underlying the anti-cancer effect of musk remain incompletely understood. Lung cancer is the leading cause of cancer-related death in the word, and the lung cancer treatment is a large problem, for example, the overall 5-year survival rate of lung cancer has merely improved from 12 to 16% over the recent 3 decades. Here, we focused on mechanisms of the growth repression and the apoptosis induced by musk in lung cancer cells. Microarray analysis showed that numerous genes were differentially expressed in Eplc-32M1 and XL-JT after native musk treatment. These differentially expressed genes were involved in many signalling pathways. The pathways which may be responsible for the growth repression and the apoptosis induced by native musk in lung cancer included interleukin family, TNF family, MAPK signalling pathway, p53 signalling pathway, and Jak-STAT signalling pathway.

Furthermore, microarray data were verified for the three genes (IL-24, interleukin family; TNFRSF25, TNF family; DDIT3, MAPK signalling pathway) by qRT-PCR in Eplc-32M1 and XL-JT treated with native musk and synthetic musk ketone. Notably, both native musk and synthetic musk ketone can induce IL-24 and DDIT3 up-regulation. IL-24, also named melanoma differentiation-associated gene-7 (mda-7) and MDA-7/IL-24, is a member of interleukin-10 family [19]. MDA-7/IL-24 acts as a growth suppressor in melanoma and other cancer cells [20]. Several studies revealed that MDA-7/IL-24 plays a key role in tumor inhibition [21–25]. TNFRSF25 (previously labelled death receptor 3) is a member of TNF family. TNFRSF25 mediates the TNFSF15- and TNFα-induced apoptosis of endothelial cells [26], and participates in the apoptosis of human osteoblasts [27]. DDIT3 belongs to MAPK signalling pathway. DDIT3 cooperates with KAT2A to up-regulate TNFRSF10A and TNFRSF10B expressions and to induce the endoplasmic reticulum stress-mediated apoptosis in lung cancer [28]. On the basis of these data and our observations, we hypothesized that musk may induce the growth repression and the apoptosis of lung cancer cell through up-regulating IL-24 and DDIT3 expressions. Musk reveals multiple biological activities. Apart from inducing the growth repression and the apoptosis of cancer cells, musk also affects immune functions. Cytokines such as interleukin and TNF, are important regulators of immune functions. We assumed that cytokine alterations caused by musk may be associated with mechanisms underlying its anti-inflammatory and immunity-enhancing effects. Mechanisms of musk action are very complex. Our research is only a pilot study, and further works are necessary.

## Conclusions

Overall, our research provided strong evidence that native musk and synthetic musk ketone can induce the growth repression and the apoptosis of cancer cells in a dose-dependent manner. However, various cancers showed significantly different responses to musk treatment. Thus, the selection of sensitive cancer patient for individualized treatment is a key step in clinical application. Synthetic musk ketone can substitute for native musk to treat cancer patients. Musk is expected to become one of adjuvant therapies for anti-cancer treatment. Additionally, musk might induce the growth repression and the apoptosis of lung cancer cells through up-regulating IL-24 and DDIT3 expressions.

## Additional files

Additional file 1: The chemical structure of synthetic musk ketone that was presented by the producer. (JPG 3 kb)
Additional file 2: The cell lines used in this study. (DOC 55 kb)
Additional file 3: Differentially expressed genes in Eplc-32M1 after treatment with native musk. (DOC 763 kb)
Additional file 4: Differentially expressed genes in XL-JT after treatment with native musk. (DOC 655 kb)

## Abbreviations

DDIT3: DNA-damage-inducible transcript 3
FCM: Flow cytometry
GC-MS: Gas chromatograph-mass spectrometer-computer
GO: Gene Ontology
IL: Interleukin
KEGG: Kyoto Encyclopedia of Genes and Genomes
MTS: 3-(4,5-dimethylthiazol-2-yl)-5-(3-carboxymethoxyphenyl)-2-(4-sulfophenyl)-2H-tetrazolium
PBS: Phosphate buffer saline
qRT-PCR: quantitative real–time polymerase chain reaction
TCM: Traditional Chinese medicine
TNF: Tumor necrosis factor
