# Supplementary figures and images for: Native musk and synthetic musk ketone strongly induced the growth repression and the apoptosis of cancer cells
Source: BMC Complement Altern Med. 2016 Dec 8;16:511. doi: 10.1186/s12906-016-1493-2 (PMC5146870; doi:10.1186/s12906-016-1493-2)

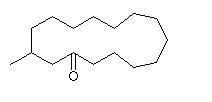

Supplement: Additional file 1: — The chemical structure of synthetic musk ketone that was presented by the producer. (JPG 3 kb) [file 12906_2016_1493_MOESM1_ESM.jpg]
