# Supplementary material for: Native musk and synthetic musk ketone strongly induced the growth repression and the apoptosis of cancer cells
Source: BMC Complement Altern Med. 2016 Dec 8;16:511. doi: 10.1186/s12906-016-1493-2 (PMC5146870; doi:10.1186/s12906-016-1493-2)
Supplement: Additional file 2: — The cell lines used in this study. (DOC 55 kb) [file 12906_2016_1493_MOESM2_ESM.doc]

**Additional file 2**. The cell lines used in this study.

| Cell type/ name | Culture media | Resource |
| --- | --- | --- |
| Lung squamous cell carcinoma |  |  |
| Eplc-32M1 | RPMI 10%FBS | German Cancer Research Center |
| Lung adenocarcinoma |  |  |
| GLC-82 | RPMI 10%FBS | Cell Bank of Chinese Academy of Science |
| XLA-07 | RPMI 10%FBS | Kunming Medical College, Kunming, China |
| XL-JT | RPMI 10%FBS | Kunming Medical College, Kunming, China |
| A549 | RPMI 10%FBS | American Type Culture Collection |
| Lung large cell carcinoma |  |  |
| NCIH-460 | RPMI 10%FBS | Gifted by Prof. Zhou Guang Biao, Beijing |
| 801-D | RPMI 10%FBS | Cell Bank of Chinese Academy of Science |
| Lung small cell carcinoma |  |  |
| NCIH-446 | RPMI 10%FBS | American Type Culture Collection |
| Mammary carcinoma |  |  |
| MDA-MB-231 | DMEM 10%FBS | American Type Culture Collection |
| MDA-MB-435 | DMEM 10%FBS | American Type Culture Collection |
| MCF-7 | DMEM 10%FBS | Conservation Genetics CAS Kunming cell Bank |
| Esophageal carcinoma |  |  |
| TE-1 | DMEM 10%FBS | Conservation Genetics CAS Kunming cell Bank |
| Gastric carcinoma |  |  |
| HSC | DMEM 10%FBS | American Type Culture Collection |
| NCI-N87 | DMEM 10%FBS | American Type Culture Collection |
| SGC-7901 | DMEM 10%FBS | American Type Culture Collection |
| Colorectal carcinoma |  |  |
| HT-29 | RPMI 10%FBS | Conservation Genetics CAS Kunming cell Bank |
| Caco-2 | DMEM 10%FBS | German Cancer Research Center |
| SW480 | DMEM 10%FBS | Gifted by Prof. Li Yan, Kunming |
| Hepatocellular carcinoma |  |  |
| HuH7 | DMEM 10%FBS | American Type Culture Collection |
| HepG2 | RPMI 10%FBS | American Type Culture Collection |
| Acute myelogenous leukemia |  |  |
| HL-60 | RPMI 10%FBS | American Type Culture Collection |
| B ell lymphoma |  |  |
| Daudi | RPMI 10%FBS | American Type Culture Collection |
