# Supplementary material for: Native musk and synthetic musk ketone strongly induced the growth repression and the apoptosis of cancer cells
Source: BMC Complement Altern Med. 2016 Dec 8;16:511. doi: 10.1186/s12906-016-1493-2 (PMC5146870; doi:10.1186/s12906-016-1493-2)
Supplement: Additional file 3: — Differentially expressed genes in Eplc-32M1 after treatment with native musk. (DOC 763 kb) [file 12906_2016_1493_MOESM3_ESM.doc]

**Additional file 3.** Differentially expressed genes in Eplc-32M1 after native musk treatment.

| **GeneSymbol** | **Log2( Fold change)** | | **Genbank** | **Gene titile** |
| --- | --- | --- | --- | --- |
| **Cytokine-cytokine receptor interaction** | | | | |
| **IL family** | |  |  |  |
| IL8 | | 8.2453165 | NM_000584 | interleukin 8 |
| IL24 | | 5.6088147 | NM_001185156 | interleukin 24 |
| IL1B | | 5.2925005 | NM_000576 | interleukin 1, beta |
| IL4R | | 5.183259 | AB102798 | interleukin 4 receptor |
| IL11 | | 3.945891 | NM_000641 | interleukin 11 |
| IL6 | | 3.9022264 | NM_000600 | interleukin 6 (interferon, beta 2) |
| IL1A | | 3.8423486 | NM_000575 | interleukin 1, alpha |
| IL20 | | 3.7252264 | NM_018724 | interleukin 20 |
| IL19 | | 3.697905 | NM_153758 | interleukin 19 |
| IL18RAP | | 2.1159005 | NM_003853 | interleukin 18 receptor accessory protein |
| IL3RA | | 2.0342112 | NM_002183 | interleukin 3 receptor, alpha (low affinity) |
| IL2RG | | 1.5126715 | NM_000206 | interleukin 2 receptor, gamma |
| IL1RAP | | 1.1702824 | NM_002182 | interleukin 1 receptor accessory protein |
| IL18R1 | | 1.1400051 | NM_003855 | interleukin 18 receptor 1 |
| IL6ST | | 1.4187331 | NM_002184 | interleukin 6 signal transducer (gp130, oncostatin M receptor) |
| IL23A | | 1.065875 | NM_016584 | interleukin 23, alpha subunit p19 |
| IL20RA | | -2.2620296 | NM_014432 | interleukin 20 receptor, alpha |
| **TNF family** | |  |  |  |
| TNFRSF9 | | 6.282377 | NM_001561 | tumor necrosis factor receptor superfamily, member 9 |
| TNFRSF25 | | 2.0581799 | NM_148965 | tumor necrosis factor receptor superfamily, member 25 |
| TNFSF15 | | 1.3671713 | NM_005118 | tumor necrosis factor (ligand) superfamily, member 15 |
| TNFRSF19 | | -2.639906 | NM_018647 | tumor necrosis factor receptor superfamily, member 19 |
| TNFSF10 | | -1.3267498 | NM_003810 | tumor necrosis factor (ligand) superfamily, member 10 |
| **Other families** | |  |  |  |
| VEGFC | | 3.8598404 | NM_005429 | vascular endothelial growth factor C |
| LIF | | 3.2915945 | NM_002309 | leukemia inhibitory factor (cholinergic differentiation factor) |
| CXCL11 | | 2.5379763 | NM_005409 | chemokine (C-X-C motif) ligand 11 |
| EDAR | | 2.5340443 | NM_022336 | ectodysplasin A receptor |
| CXCL10 | | 2.4921036 | NM_001565 | chemokine (C-X-C motif) ligand 10 |
| CCL5 | | 2.3072252 | NM_002985 | chemokine (C-C motif) ligand 5 |
| INHBB | | 2.1035213 | NM_002193 | inhibin, beta B |
| OSMR | | 2.1026545 | NM_001168355 | oncostatin M receptor |
| CXCL1 | | 1.7411547 | NM_001511 | chemokine (C-X-C motif) ligand 1alpha) |
| CXCL3 | | 1.6780405 | NM_002090 | chemokine (C-X-C motif) ligand 3 |
| PLEKHG5 | | 1.6512403 | NM_198681 | pleckstrin homology domain containing, family G ber 5 |
| TNS4 | | 1.5763063 | NM_032865 | tensin 4 |
| VEGFA | | 1.5091877 | NM_001025366 | vascular endothelial growth factor A |
| ACVR1 | | 1.455174 | NM_001105 | activin A receptor, type I |
| BMP2 | | 1.2659893 | NM_001200 | bone morphogenetic protein 2 |
| CXCL2 | | 1.2573347 | NM_002089 | chemokine (C-X-C motif) ligand 2 |
| XLOC_l2_015148 | | 1.2514534 | XR_108937 |  |
| FLJ44511 | | 1.124855 | NR_033963 | uncharacterized LOC441307 |
| PDGFA | | 1.0315065 | NM_033023 | platelet-derived growth factor alpha polypeptide |
| CTF1 | | 1.0183606 | NM_001330 | cardiotrophin 1 |
| TPO | | -4.1799746 | NM_175722 | thyroid peroxidase |
| BMP5 | | -3.4071732 | NM_021073 | bone morphogenetic protein 5 |
| CCL26 | | -3.0761533 | NM_006072 | chemokine (C-C motif) ligand 26 |
| AMHR2 | | -2.7227597 | NM_020547 | anti-Mullerian hormone receptor, type II |
| GDF5 | | -1.5018377 | NM_000557 | growth differentiation factor 5 |
| CXCR3 | | -1.354311 | NM_001504 | chemokine (C-X-C motif) receptor 3 |
| PGF | | -1.1488256 | NM_002632 | placental growth factor |
| LOC283888 | | -1.0934653 | NR_037158 | uncharacterized LOC283888 |
| LIFR | | -1.068594 | NM_002310 | leukemia inhibitory factor receptor alpha |
| **Jak-STAT signaling pathway** | | | | |
| IL24 | | 5.6088147 | NM_001185156 | interleukin 24 |
| IL4R | | 5.183259 | AB102798 | interleukin 4 receptor |
| IL11 | | 3.945891 | NM_000641 | interleukin 11 |
| IL6 | | 3.9022264 | NM_000600 | interleukin 6 (interferon, beta 2) |
| SPRY4 | | 3.727611 | NM_030964 | sprouty homolog 4 (Drosophila) |
| IL20 | | 3.7252264 | NM_018724 | interleukin 20 |
| IL19 | | 3.697905 | NM_153758 | interleukin 19 |
| LIF | | 3.2915945 | NM_002309 | leukemia inhibitory factor (cholinergic differentiation factor) |
| SPRED2 | | 3.0955615 | NM_181784 | sprouty-related, EVH1 domain containing 2 |
| IL13RA2 | | 2.9528522 | NM_000640 | interleukin 13 receptor, alpha 2 |
| OSMR | | 2.1026545 | NM_001168355 | oncostatin M receptor |
| IL3RA | | 2.0342112 | NM_002183 | interleukin 3 receptor, alpha (low affinity) |
| SH3YL1 | | 1.691999 | NM_015677 | SH3 domain containing, Ysc84-like 1 (S. cerevisiae) |
| PIM1 | | 1.5444288 | NM_002648 | pim-1 oncogene |
| IL2RG | | 1.5126715 | NM_000206 | interleukin 2 receptor, gamma |
| IL6ST | | 1.4187331 | NM_002184 | interleukin 6 signal transducer (gp130, oncostatin M receptor) |
| CCND1 | | 1.3763199 | NM_053056 | cyclin D1 |
| SPRED1 | | 1.3759413 | NM_152594 | sprouty-related, EVH1 domain containing 1 |
| CBLB | | 1.2378068 | NM_170662 | Cas-Br-M (murine) ecotropic retroviral transforming sequence b |
| PIK3CD | | 1.2172623 | NM_005026 | phosphoinositide-3-kinase, catalytic, delta polypeptide |
| SOS2 | | 1.0677557 | NM_006939 | son of sevenless homolog 2 (Drosophila) |
| IL23A | | 1.065875 | NM_016584 | interleukin 23, alpha subunit p19 |
| JAK2 | | 1.043479 | NM_004972 | Janus kinase 2 |
| CTF1 | | 1.0183606 | NM_001330 | cardiotrophin 1 |
| SOCS3 | | 1.0093479 | NM_003955 | suppressor of cytokine signaling 3 |
| TPO | | -4.1799746 | NM_175722 | thyroid peroxidase |
| IL20RA | | -2.2620296 | NM_014432 | interleukin 20 receptor, alpha |
| AKT3 | | -2.0072546 | NM_181690 | v-akt murine thymoma viral oncogene homolog 3 |
| MAP7D3 | | -1.1497521 | NM_001173517 | MAP7 domain containing 3 |
| NOSTRIN | | -1.0960379 | NM_052946 | nitric oxide synthase trafficker |
| LIFR | | -1.068594 | NM_002310 | leukemia inhibitory factor receptor alpha |
| **p53 signaling pathway** | | | | |
| IGFBP1 | | 4.066637 | NM_000596 | insulin-like growth factor binding protein 1 |
| CDKN1A | | 2.8123603 | NM_078467 | cyclin-dependent kinase inhibitor 1A (p21, Cip1) |
| SERPINE1 | | 1.8331661 | NM_000602 | serpin peptidase inhibitor, clade E |
| TSPAN19 | | 1.8122759 | NM_001100917 | tetraspanin 19 |
| RALGAPA2 | | 1.7687001 | NM_020343 | Ral GTPase activating protein, alpha subunit 2 (catalytic) |
| STEAP2 | | 1.6648774 | NM_152999 | STEAP family member 2, metalloreductase |
| SESN2 | | 1.4196215 | NM_031459 | sestrin 2 |
| CCND1 | | 1.3763199 | NM_053056 | cyclin D1 |
| IGFBP4 | | 1.3470583 | NM_001552 | insulin-like growth factor binding protein 4 |
| STEAP1 | | 1.1124754 | NM_012449 | six transmembrane epithelial antigen of the prostate 1 |
| MDM4 | | 1.0825663 | NM_002393 | Mdm4 p53 binding protein homolog (mouse) |
| TNS1 | | -1.3698735 | NM_022648 | tensin 1 |
| BAI1 | | -1.1565113 | NM_001702 | brain-specific angiogenesis inhibitor 1 |
| TNS3 | | -1.1107836 | NM_022748 | tensin 3 |
| **MAPK signaling pathway** | | | | |
| IL1B | | 5.2925005 | NM_000576 | interleukin 1, beta |
| DUSP6 | | 4.377489 | NM_001946 | dual specificity phosphatase 6 |
| IL1A | | 3.8423486 | NM_000575 | interleukin 1, alpha |
| CACNG6 | | 3.672966 | NM_145814 | calcium channel, voltage-dependent, gamma subunit 6 |
| FOSL1 | | 3.4074564 | NM_005438 | FOS-like antigen 1 |
| DUSP5 | | 3.3937073 | NM_004419 | dual specificity phosphatase 5 |
| RELB | | 3.0128002 | NM_006509 | v-rel reticuloendotheliosis viral oncogene homolog B |
| IKBKB | | 2.7367592 | AK309052 | inhibitor of kappa light polypeptide gene enhancer in B-cells |
| DUSP4 | | 2.66405 | NM_001394 | dual specificity phosphatase 4 |
| RCAN2 | | 2.494337 | NM_001251973 | regulator of calcineurin 2 |
| FOS | | 2.0178242 | NM_005252 | FBJ murine osteosarcoma viral oncogene homolog |
| JUNB | | 1.9544878 | NM_002229 | jun B proto-oncogene |
| BDNF | | 1.8525515 | NM_170735 | brain-derived neurotrophic factor |
| JUN | | 1.8334875 | NM_002228 | jun proto-oncogene |
| STMN3 | | 1.7885718 | NM_015894 | stathmin-like 3 |
| DDIT3 | | 1.75354 | NM_004083 | DNA-damage-inducible transcript 3 |
| SH3YL1 | | 1.691999 | NM_015677 | SH3 domain containing, Ysc84-like 1 (S. cerevisiae) |
| RASGRP2 | | 1.6811047 | NM_153819 | RAS guanyl releasing protein 2 (calcium and DAG-regulated) |
| MCTP1 | | 1.5820065 | NM_024717 | multiple C2 domains, transmembrane 1 |
| ZC3H12C | | 1.5367727 | NM_033390 | zinc finger CCCH-type containing 12C |
| TESC | | 1.5329237 | NM_017899 | tescalcin |
| PTPRR | | 1.4828339 | NM_002849 | protein tyrosine phosphatase, receptor type, R |
| NFKB2 | | 1.4605198 | NM_001077493 | nuclear factor of kappa light polypeptide gene enhancer in B-cells 2) |
| FOSB | | 1.4260874 | NM_006732 | FBJ murine osteosarcoma viral oncogene homolog B |
| MAP3K2 | | 1.3535337 | NM_006609 | mitogen-activated protein kinase kinase kinase 2 |
| CDC42EP2 | | 1.3375244 | NM_006779 | CDC42 effector protein (Rho GTPase binding) 2 |
| FLJ44511 | | 1.124855 | NR_033963 | uncharacterized LOC441307 |
| CACNG8 | | 1.0947104 | NM_031895 | calcium channel, voltage-dependent, gamma subunit 8 |
| SOS2 | | 1.0677557 | NM_006939 | son of sevenless homolog 2 (Drosophila) |
| TAOK1 | | 1.0422497 | NM_020791 | TAO kinase 1 |
| PDGFA | | 1.0315065 | NM_033023 | platelet-derived growth factor alpha polypeptide |
| JUND | | 1.0222626 | NM_005354 | jun D proto-oncogene |
| PRKCB | | -6.210244 | NM_002738 | protein kinase C, beta |
| FGFR2 | | -5.07627 | NM_022970 | fibroblast growth factor receptor 2 |
| FGF13 | | -2.8540053 | NM_004114 | fibroblast growth factor 13 |
| HSPA2 | | -2.5928597 | NM_021979 | heat shock 70kDa protein 2 |
| CACNA1B | | -2.5504093 | NM_000718 | calcium channel, voltage-dependent, N type, alpha 1B subunit |
| MAP2K6 | | -2.4668016 | NM_002758 | mitogen-activated protein kinase kinase 6 |
| CACNB2 | | -2.2987142 | NM_000724 | calcium channel, voltage-dependent, beta 2 subunit |
| AKT3 | | -2.0072546 | NM_181690 | v-akt murine thymoma viral oncogene homolog 3 |
| CDC42BPG | | -1.4571857 | NM_017525 | CDC42 binding protein kinase gamma (DMPK-like) |
| RASAL2 | | -1.3561573 | NM_170692 | RAS protein activator like 2 |
| DUSP14 | | -1.1880131 | NM_007026 | dual specificity phosphatase 14 |
| PLA2G1B | | -1.1807537 | NM_000928 | phospholipase A2, group IB (pancreas) |
| CDC42 | | -1.1461239 | NM_044472 | cell division cycle 42 (GTP binding protein, 25kDa) |
| DUSP9 | | -1.1361408 | NM_001395 | dual specificity phosphatase 9 |
| NOSTRIN | | -1.0960379 | NM_052946 | nitric oxide synthase trafficker |
| MAPK10 | | -1.0286255 | NM_138980 | mitogen-activated protein kinase 10 |
| **Alanine, aspartate and glutamate metabolism** | | | | |
| ABAT | | 3.5672412 | NM_000663 | 4-aminobutyrate aminotransferase |
| GFPT1 | | 1.2575054 | NM_001244710 | glutamine--fructose-6-phosphate transaminase 1 |
| LGSN | | -3.8157363 | NM_016571 | lengsin, lens protein with glutamine synthetase domain |
| ACY3 | | -2.5407052 | NM_080658 | aspartoacylase (aminocyclase) 3 |
| ALDH4A1 | | -1.5408459 | NM_170726 | aldehyde dehydrogenase 4 family, member A1 |
| IL4I1 | | -1.0236831 | NM_172374 | interleukin 4 induced 1 |
| ALDH5A1 | | -1.0157614 | NM_170740 | aldehyde dehydrogenase 5 family, member A1 |
| **Nicotinate and nicotinamide metabolism** | | | | |
| NT5E | | 3.8185692 | NM_002526 | 5'-nucleotidase, ecto (CD73) |
| NMNAT2 | | 2.0591583 | NM_015039 | nicotinamide nucleotide adenylyltransferase 2 |
| NAMPT | | 1.8069019 | NM_005746 | nicotinamide phosphoribosyltransferase |
| NT5M | | 1.7217379 | NM_020201 | 5',3'-nucleotidase, mitochondrial |
| ENPP1 | | -1.3390284 | NM_006208 | ectonucleotide pyrophosphatase/phosphodiesterase 1 |
| **Osteoclast differentiation** | | | | |
| IL1B | | 5.2925005 | NM_000576 | interleukin 1, beta |
| IL1A | | 3.8423486 | NM_000575 | interleukin 1, alpha |
| FOSL1 | | 3.4074564 | NM_005438 | FOS-like antigen 1 |
| RELB | | 3.0128002 | NM_006509 | v-rel reticuloendotheliosis viral oncogene homolog B |
| IKBKB | | 2.7367592 | AK309052 | inhibitor of kappa light polypeptide gene enhancer in B-cells |
| FOS | | 2.0178242 | NM_005252 | FBJ murine osteosarcoma viral oncogene homolog |
| JUNB | | 1.9544878 | NM_002229 | jun B proto-oncogene |
| JUN | | 1.8334875 | NM_002228 | jun proto-oncogene |
| SH3YL1 | | 1.691999 | NM_015677 | SH3 domain containing, Ysc84-like 1 (S. cerevisiae) |
| GAB3 | | 1.6752939 | NM_080612 | GRB2-associated binding protein 3 |
| NFKB2 | | 1.4605198 | NM_001077493 | nuclear factor of kappa light polypeptide gene enhancer in B-cells 2 |
| FOSB | | 1.4260874 | NM_006732 | FBJ murine osteosarcoma viral oncogene homolog B |
| OSCAR | | 1.3980045 | NM_206818 | osteoclast associated, immunoglobulin-like receptor |
| CTSK | | 1.3623104 | NM_000396 | cathepsin K |
| PIK3CD | | 1.2172623 | NM_005026 | phosphoinositide-3-kinase, catalytic, delta polypeptide |
| PPARG | | 1.2038794 | NM_138711 | peroxisome proliferator-activated receptor gamma |
| NCF2 | | 1.0901747 | NM_000433 | neutrophil cytosolic factor 2 |
| SH3PXD2B | | 1.0339966 | NM_001017995 | SH3 and PX domains 2B |
| JUND | | 1.0222626 | NM_005354 | jun D proto-oncogene |
| SOCS3 | | 1.0093479 | NM_003955 | suppressor of cytokine signaling 3 |
| NOX4 | | -4.229009 | NM_001143836 | NADPH oxidase 4 |
| MAP2K6 | | -2.4668016 | NM_002758 | mitogen-activated protein kinase kinase 6 |
| AKT3 | | -2.0072546 | NM_181690 | v-akt murine thymoma viral oncogene homolog 3 |
| MITF | | -1.8201065 | NM_198159 | microphthalmia-associated transcription factor |
| MAP7D3 | | -1.1497521 | NM_001173517 | MAP7 domain containing 3 |
| NOSTRIN | | -1.0960379 | NM_052946 | nitric oxide synthase trafficker |
| MAPK10 | | -1.0286255 | NM_138980 | mitogen-activated protein kinase 10 |
| **Retinol metabolism** | | | | |
| CYP1A1 | | 3.4747272 | NM_000499 | cytochrome P450, family 1, subfamily A, polypeptide 1 |
| CYP26B1 | | 2.8316493 | NM_019885 | cytochrome P450, family 26, subfamily B, polypeptide 1 |
| DHRS3 | | 1.8403339 | NM_004753 | dehydrogenase/reductase (SDR family) member 3 |
| SDR16C5 | | 1.359088 | NM_138969 | short chain dehydrogenase/reductase family 16C, member 5 |
| CYP3A4 | | 1.1207728 | NM_017460 | cytochrome P450, family 3, subfamily A, polypeptide 4 |
| HSD17B13 | | -4.343038 | NM_178135 | hydroxysteroid (17-beta) dehydrogenase 13 |
| ALDH1A2 | | -2.5922751 | NM_170697 | aldehyde dehydrogenase 1 family, member A2 |
| ALDH1A1 | | -2.552958 | NM_000689 | aldehyde dehydrogenase 1 family, member A1 |
| RDH12 | | -1.5311174 | NM_152443 | retinol dehydrogenase 12 (all-trans/9-cis/11-cis) |
| PNPLA4 | | -1.5025587 | NM_004650 | patatin-like phospholipase domain containing 4 |
| HRASLS2 | | -1.0500278 | NM_017878 | HRAS-like suppressor 2 |
| **Rheumatoid arthritis** | | | | |
| MMP1 | | 9.23877 | NM_002421 | matrix metallopeptidase 1 (interstitial collagenase) |
| IL8 | | 8.2453165 | NM_000584 | interleukin 8 |
| IL1B | | 5.2925005 | NM_000576 | interleukin 1, beta |
| IL11 | | 3.945891 | NM_000641 | interleukin 11 |
| IL6 | | 3.9022264 | NM_000600 | interleukin 6 (interferon, beta 2) |
| IL1A | | 3.8423486 | NM_000575 | interleukin 1, alpha |
| FOSL1 | | 3.4074564 | NM_005438 | FOS-like antigen 1 |
| ATP6V0D2 | | 3.0207453 | NM_152565 | ATPase, H+ transporting, lysosomal 38kDa, V0 subunit d2 |
| CCL5 | | 2.3072252 | NM_002985 | chemokine (C-C motif) ligand 5 |
| FOS | | 2.0178242 | NM_005252 | FBJ murine osteosarcoma viral oncogene homolog |
| ATP6AP1L | | 1.9716043 | NM_001017971 | ATPase, H+ transporting, lysosomal accessory protein 1-like |
| JUNB | | 1.9544878 | NM_002229 | jun B proto-oncogene |
| ICAM1 | | 1.9410534 | NM_000201 | intercellular adhesion molecule 1 |
| HLA-DMB | | 1.853116 | NM_002118 | major histocompatibility complex, class II, DM beta |
| JUN | | 1.8334875 | NM_002228 | jun proto-oncogene |
| LOC497256 | | 1.764287 | AK094988 | uncharacterized LOC497256 |
| TLR4 | | 1.5272398 | NM_138554 | toll-like receptor 4 |
| VEGFA | | 1.5091877 | NM_001025366 | vascular endothelial growth factor A |
| FOSB | | 1.4260874 | NM_006732 | FBJ murine osteosarcoma viral oncogene homolog B |
| IL6ST | | 1.4187331 | NM_002184 | interleukin 6 signal transducer (gp130, oncostatin M receptor) |
| CTSL1P8 | | 1.385272 | NR_033405 | cathepsin L1 pseudogene 8 |
| CTSK | | 1.3623104 | NM_000396 | cathepsin K |
| MMP14 | | 1.2412271 | NM_004995 | matrix metallopeptidase 14 (membrane-inserted) |
| CTSL1P2 | | 1.1278696 | NR_033407 | cathepsin L1 pseudogene 2 |
| CTSL1 | | 1.0701418 | NM_001912 | cathepsin L1 |
| IL23A | | 1.065875 | NM_016584 | interleukin 23, alpha subunit p19 |
| JUND | | 1.0222626 | NM_005354 | jun D proto-oncogene |
| ATP6V1G3 | | -2.4130583 | NM_133326 | ATPase, H+ transporting, lysosomal 13kDa, V1 subunit G3 |
| PGF | | -1.1488256 | NM_002632 | placental growth factor |
| **Steroid hormone biosynthesis** | | | | |
| CYP19A1 | | 3.8985023 | NM_031226 | cytochrome P450, family 19, subfamily A, polypeptide 1 |
| CYP1A1 | | 3.4747272 | NM_000499 | cytochrome P450, family 1, subfamily A, polypeptide 1 |
| CYP1B1 | | 2.3188448 | NM_000104 | cytochrome P450, family 1, subfamily B, polypeptide 1 |
| STS | | 2.034235 | NM_000351 | steroid sulfatase (microsomal), isozyme S |
| HSD17B14 | | 1.4878764 | NM_016246 | hydroxysteroid (17-beta) dehydrogenase 14 |
| HSD17B7 | | 1.3135004 |  | hydroxysteroid (17-beta) dehydrogenase 7 |
| CYP3A4 | | 1.1207728 | NM_017460 | cytochrome P450, family 3, subfamily A, polypeptide 4 |
| SULT2B1 | | -3.48801 | NM_004605 | sulfotransferase family, cytosolic, 2B, member 1 |
| AKR1C4 | | -3.244865 | NM_001818 | aldo-keto reductase family 1, member C4 |
| SRD5A3 | | -1.3889313 | NM_024592 | steroid 5 alpha-reductase 3 |
| CYP19A1 | | -1.3518972 | NM_031226 | cytochrome P450, family 19, subfamily A, polypeptide 1 |
| **ABC transporters** | | | | |
| ABCG1 | | 1.3316908 | NM_207627 | ATP-binding cassette, sub-family G (WHITE), member 1 |
| ABCA13 | | -5.85645 | NM_152701 | ATP-binding cassette, sub-family A (ABC1), member 13 |
| CFTR | | -2.25495 | NM_000492 | cystic fibrosis transmembrane conductance regulator |
| ABCB9 | | -2.0015206 | AK027624 | ATP-binding cassette, sub-family B (MDR/TAP), member 9 |
| ABCC6 | | -1.5210562 | NM_001079528 | ATP-binding cassette, sub-family C (CFTR/MRP), member 6 |
| ABCD3 | | -1.4811716 | NM_001122674 | ATP-binding cassette, sub-family D (ALD), member 3 |
| ABCB10 | | -1.4416618 | NM_012089 | ATP-binding cassette, sub-family B (MDR/TAP), member 10 |
| ABCB8 | | -1.2624454 | NM_007188 | ATP-binding cassette, sub-family B (MDR/TAP), member 8 |
| ABCD1 | | -1.2617741 | NM_000033 | ATP-binding cassette, sub-family D (ALD), member 1 |
| ABCC5 | | -1.0290308 | NM_001023587 | ATP-binding cassette, sub-family C (CFTR/MRP), member 5 |
| **Arrhythmogenic right ventricular cardiomyopathy (ARVC)** | | | | |
| CACNG6 | | 3.672966 | NM_145814 | calcium channel, voltage-dependent, gamma subunit 6 |
| ITGB7 | | 3.234314 | NM_000889 | integrin, beta 7 |
| ITGA2 | | 2.6736517 | NM_002203 | integrin, alpha 2 (CD49B, alpha 2 subunit of VLA-2 receptor) |
| CLMN | | 2.3493013 | NM_024734 | calmin (calponin-like, transmembrane) |
| ITGB4 | | 2.0468588 | NM_000213 | integrin, beta 4 |
| IFLTD1 | | 2.0200586 | NM_001145729 | intermediate filament tail domain containing 1 |
| ITGA6 | | 1.9236512 | NM_000210 | integrin, alpha 6 |
| C14orf49 | | 1.6756778 | NM_152592 | chromosome 14 open reading frame 49 |
| ITGA1 | | 1.2995157 | NM_181501 | integrin, alpha 1 |
| DMD | | 1.2630501 | NM_004021 | dystrophin |
| TCF7L2 | | 1.2279196 | NM_030756 | transcription factor 7-like 2 (T-cell specific, HMG-box) |
| CACNG8 | | 1.0947104 | NM_031895 | calcium channel, voltage-dependent, gamma subunit 8 |
| TCF7L1 | | 1.0414724 | NM_031283 | transcription factor 7-like 1 (T-cell specific, HMG-box) |
| RYR2 | | -4.693291 | NM_001035 | ryanodine receptor 2 (cardiac) |
| ACTL8 | | -2.6115284 | NM_030812 | actin-like 8 |
| CTNNA2 | | -2.4047928 | NM_004389 | catenin (cadherin-associated protein), alpha 2 |
| CACNB2 | | -2.2987142 | NM_000724 | calcium channel, voltage-dependent, beta 2 subunit |
| ITGBL1 | | -2.2509308 | NM_004791 | integrin, beta-like 1 (with EGF-like repeat domains) |
| MICALCL | | -1.4798837 | NM_032867 | MICAL C-terminal like |
| DES | | -1.014029 | NM_001927 | desmin |
| **Axon guidance** | | | | |
| RGS17 | | 3.3739238 | NM_012419 | regulator of G-protein signaling 17 |
| MDGA1 | | 3.0623078 | NM_153487 | MAM domain containing glycosylphosphatidylinositol anchor 1 |
| AMIGO2 | | 2.8145962 | NM_181847 | adhesion molecule with Ig-like domain 2 |
| LOC100289495 | | 2.6865034 | NR_040022 | uncharacterized LOC100289495 |
| SEMA4B | | 2.6455836 | NM_020210 | sema domain, immunoglobulin domain (Ig) |
| ABLIM2 | | 2.594613 | NM_001130083 | actin binding LIM protein family, member 2 |
| ABLIM3 | | 2.507617 | NM_014945 | actin binding LIM protein family, member 3 |
| SHB | | 2.060567 | NM_003028 | Src homology 2 domain containing adaptor protein B |
| UNC5B | | 1.9215488 | NM_170744 | unc-5 homolog B (C. elegans) |
| RGS3 | | 1.8847275 | NM_134427 | regulator of G-protein signaling 3 |
| TPBG | | 1.6783867 | NM_006670 | trophoblast glycoprotein |
| EFNB2 | | 1.6157122 | NM_004093 | ephrin-B2 |
| TESC | | 1.5329237 | NM_017899 | tescalcin |
| RGS16 | | 1.4353046 | NM_002928 | regulator of G-protein signaling 16 |
| EPHA2 | | 1.3777971 | NM_004431 | EPH receptor A2 |
| CDC42EP2 | | 1.3375244 | NM_006779 | CDC42 effector protein (Rho GTPase binding) 2 |
| NRP1 | | 1.3081341 | NM_003873 | neuropilin 1 |
| SLIT2 | | 1.1033306 | NM_004787 | slit homolog 2 (Drosophila) |
| LINGO2 | | 1.0362015 | NM_152570 | leucine rich repeat and Ig domain containing 2 |
| NTN4 | | 1.0239172 | NM_021229 | netrin 4 |
| EPHA7 | | -3.861567 | NM_004440 | EPH receptor A7 |
| SLIT1 | | -3.056584 | NM_003061 | slit homolog 1 (Drosophila) |
| SEMA3C | | -2.6301146 | NM_006379 | sema domain, immunoglobulin domain (Ig), short basic domain, |
| ITGBL1 | | -2.2509308 | NM_004791 | integrin, beta-like 1 (with EGF-like repeat domains) |
| EPHA3 | | -2.243671 | NM_005233 | EPH receptor A3 |
| DPYSL4 | | -1.7330413 | NM_006426 | dihydropyrimidinase-like 4 |
| UNC5A | | -1.5133023 | NM_133369 | unc-5 homolog A (C. elegans) |
| CDC42BPG | | -1.4571857 | NM_017525 | CDC42 binding protein kinase gamma (DMPK-like) |
| RASAL2 | | -1.3561573 | NM_170692 | RAS protein activator like 2 |
| VWDE | | -1.2905269 | NM_001135924 | von Willebrand factor D and EGF domains |
| SLITRK6 | | -1.2683592 | NM_032229 | SLIT and NTRK-like family, member 6 |
| CFL2 | | -1.165699 | NM_021914 | cofilin 2 (muscle) |
| CDC42 | | -1.1461239 | NM_044472 | cell division cycle 42 (GTP binding protein, 25kDa) |
| NFATC1 | | -1.0985823 | NM_172387 | nuclear factor of activated T-cells |
| **Complement and coagulation cascades** | | | | |
| PLAT | | 4.045933 | NM_000930 | plasminogen activator, tissue |
| C1S | | 3.5531564 | NM_201442 | complement component 1, s subcomponent |
| CR2 | | 3.105011 | NM_001006658 | complement component (3d/Epstein Barr virus) receptor 2 |
| BDKRB1 | | 2.2677045 | NM_000710 | bradykinin receptor B1 |
| C5orf41 | | 2.0115957 | NM_153607 | chromosome 5 open reading frame 41 |
| C5 | | 1.866108 | NM_001735 | complement component 5 |
| SERPINE1 | | 1.8331661 | NM_000602 | serpin peptidase inhibitor, clade E |
| EDIL3 | | 1.7876105 | NM_005711 | EGF-like repeats and discoidin I-like domains 3 |
| C5orf49 | | 1.7493181 | NM_001089584 | chromosome 5 open reading frame 49 |
| C4BPA | | 1.5727797 | NM_000715 | complement component 4 binding protein, alpha |
| TFPI2 | | 1.5282154 | NM_006528 | tissue factor pathway inhibitor 2 |
| C4B | | 1.3561616 | NM_001002029 | complement component 4B (Chido blood group) |
| LOC390940 | | 1.2625546 | NM_001193621 | uncharacterized protein ENSP00000244321 |
| PLAUR | | 1.2355595 | NM_001005377 | plasminogen activator, urokinase receptor |
| PRRG1 | | 1.1348982 | NM_001173486 | proline rich Gla (G-carboxyglutamic acid) 1 |
| C1RL | | 1.0812883 | NM_016546 | complement component 1, r subcomponent-like |
| TFPI | | 1.0180941 | NM_001032281 | tissue factor pathway inhibitor |
| OTOG | | -3.211523 | XM_001717531 | otogelin |
| LOC100289038 | | -3.1103573 | XR_132906 | von Willebrand factor-like |
| VWF | | -2.6140704 | NM_000552 | von Willebrand factor |
| C5orf42 | | -2.250946 | NM_023073 | chromosome 5 open reading frame 42 |
| F10 | | -2.2038984 | NM_000504 | coagulation factor X |
| KREMEN1 | | -1.6943202 | NM_001039570 | kringle containing transmembrane protein 1 |
| C5orf13 | | -1.455802 | NM_001142483 | chromosome 5 open reading frame 13 |
| CBLN2 | | -1.2003975 | NM_182511 | cerebellin 2 precursor |
| CFI | | -1.1751809 | NM_000204 | complement factor I |
| CBLN4 | | -1.0813103 | NM_080617 | cerebellin 4 precursor |
| F2 | | -1.0689635 | NM_000506 | coagulation factor II (thrombin) |
| CFHR3 | | -1.0212002 | NM_021023 | complement factor H-related 3 |
| **Dilated cardiomyopathy** | | | | |
| CACNG6 | | 3.672966 | NM_145814 | calcium channel, voltage-dependent, gamma subunit 6 |
| ADRB1 | | 3.4933672 | NM_000684 | adrenergic, beta-1-, receptor |
| ITGB7 | | 3.234314 | NM_000889 | integrin, beta 7 |
| ITGA2 | | 2.6736517 | NM_002203 | integrin, alpha 2 (CD49B, alpha 2 subunit of VLA-2 receptor) |
| ADCY6 | | 2.575365 | NM_015270 | adenylate cyclase 6 |
| ITGB4 | | 2.0468588 | NM_000213 | integrin, beta 4 |
| IFLTD1 | | 2.0200586 | NM_001145729 | intermediate filament tail domain containing 1 |
| ITGA6 | | 1.9236512 | NM_000210 | integrin, alpha 6 |
| ITGA1 | | 1.2995157 | NM_181501 | integrin, alpha 1 |
| DMD | | 1.2630501 | NM_004021 | dystrophin |
| CACNG8 | | 1.0947104 | NM_031895 | calcium channel, voltage-dependent, gamma subunit 8 |
| OBSCN | | 1.0740819 | BC043916 | obscurin, cytoskeletal calmodulin and titin-interacting RhoGEF |
| RYR2 | | -4.5727572 | NM_001035 | ryanodine receptor 2 (cardiac) |
| ACTL8 | | -2.6115284 | NM_030812 | actin-like 8 |
| CACNB2 | | -2.2987142 | NM_000724 | calcium channel, voltage-dependent, beta 2 subunit |
| ITGBL1 | | -2.2509308 | NM_004791 | integrin, beta-like 1 (with EGF-like repeat domains) |
| DES | | -1.014029 | NM_001927 | desmin |
| **ECM-receptor interaction** | | | | |
| ITGB7 | | 3.234314 | NM_000889 | integrin, beta 7 |
| ITGA2 | | 2.6736517 | NM_002203 | integrin, alpha 2 (CD49B, alpha 2 subunit of VLA-2 receptor) |
| LAMB2P1 | | 2.211647 | NR_004405 | laminin, beta 2 pseudogene 1 |
| LAMA4 | | 2.1986122 | NM_001105206 | laminin, alpha 4 |
| ITGB4 | | 2.0468588 | NM_000213 | integrin, beta 4 |
| ITGA6 | | 1.9236512 | NM_000210 | integrin, alpha 6 |
| FLJ21408 | | 1.7384233 | AK025061 | uncharacterized LOC400512 |
| LAMC2 | | 1.5982022 | NM_018891 | laminin, gamma 2 |
| SDC4 | | 1.4799623 | NM_002999 | syndecan 4 |
| CD44 | | 1.4527559 | NM_001202557 | CD44 molecule (Indian blood group) |
| ITGA1 | | 1.2995157 | NM_181501 | integrin, alpha 1 |
| COL4A1 | | 1.0665026 | NM_001845 | collagen, type IV, alpha 1 |
| OTOG | | -3.211523 | XM_001717531 | otogelin |
| LOC100289038 | | -3.1103573 | XR_132906 | von Willebrand factor-like |
| VWF | | -2.6140704 | NM_000552 | von Willebrand factor |
| SV2B | | -2.5764847 | NM_014848 | synaptic vesicle glycoprotein 2B |
| TMEFF2 | | -2.4207911 | NM_016192 | transmembrane protein with EGF-like and two follistatin-like  domains 2 |
| ITGBL1 | | -2.2509308 | NM_004791 | integrin, beta-like 1 (with EGF-like repeat domains) |
| COL24A1 | | -1.5017715 | NM_152890 | collagen, type XXIV, alpha 1 |
| SPINK5 | | -1.4005194 | NM_001127698 | serine peptidase inhibitor, Kazal type 5 |
| CD36 | | -1.30968 | NM_001001547 | CD36 molecule (thrombospondin receptor) |
| VWA2 | | -1.1014357 | NM_198496 | von Willebrand factor A domain containing 2 |
| CHADL | | -1.0455093 | NM_138481 | chondroadherin-like |
| **Epithelial cell signaling in Helicobacter pylori infection** | | | | |
| IL8 | | 8.2453165 | NM_000584 | interleukin 8 |
| ATP6V0D2 | | 3.0207453 | NM_152565 | ATPase, H+ transporting, lysosomal 38kDa, V0 subunit d2 |
| IKBKB | | 2.7367592 | AK309052 | inhibitor of kappa light polypeptide gene enhancer in B-cells |
| CCL5 | | 2.3072252 | NM_002985 | chemokine (C-C motif) ligand 5 |
| ATP6AP1L | | 1.9716043 | NM_001017971 | ATPase, H+ transporting, lysosomal accessory protein 1-like |
| JUNB | | 1.9544878 | NM_002229 | jun B proto-oncogene |
| JUN | | 1.8334875 | NM_002228 | jun proto-oncogene |
| CDC42EP2 | | 1.3375244 | NM_006779 | CDC42 effector protein (Rho GTPase binding) 2 |
| ADAM17 | | 1.1799164 | NM_003183 | ADAM metallopeptidase domain 17 |
| JUND | | 1.0222626 | NM_005354 | jun D proto-oncogene |
| ATP6V1G3 | | -2.4130583 | NM_133326 | ATPase, H+ transporting, lysosomal 13kDa, V1 subunit G3 |
| JAM3 | | -1.5630703 | NM_032801 | junctional adhesion molecule 3 |
| CDC42BPG | | -1.4571857 | NM_017525 | CDC42 binding protein kinase gamma (DMPK-like) |
| NLRC5 | | -1.2417803 | NM_032206 | NLR family, CARD domain containing 5 |
| CDC42 | | -1.1461239 | NM_044472 | cell division cycle 42 (GTP binding protein, 25kDa) |
| JAM2 | | -1.0542111 | AK056079 | junctional adhesion molecule 2 |
| MAPK10 | | -1.0286255 | NM_138980 | mitogen-activated protein kinase 10 |
| LRRC45 | | -1.0104465 | NM_144999 | leucine rich repeat containing 45 |
| **ErbB signaling pathway** | | | | |
| SHC4 | | 4.224395 | NM_203349 | SHC (Src homology 2 domain containing) family, member 4 |
| AREG | | 3.9725513 | NM_001657 | amphiregulin |
| EPGN | | 3.6440978 | NM_001013442 | epithelial mitogen homolog (mouse) |
| CDKN1A | | 2.8123603 | NM_078467 | cyclin-dependent kinase inhibitor 1A (p21, Cip1) |
| EREG | | 2.202065 | NM_001432 | epiregulin |
| SHB | | 2.060567 | NM_003028 | Src homology 2 domain containing adaptor protein B |
| JUNB | | 1.9544878 | NM_002229 | jun B proto-oncogene |
| JUN | | 1.8334875 | NM_002228 | jun proto-oncogene |
| SH3YL1 | | 1.691999 | NM_015677 | SH3 domain containing, Ysc84-like 1 (S. cerevisiae) |
| MCTP1 | | 1.5820065 | NM_024717 | multiple C2 domains, transmembrane 1 |
| TGFA | | 1.5458126 | NM_003236 | transforming growth factor, alpha |
| NCK2 | | 1.3113194 | NM_003581 | NCK adaptor protein 2 |
| CBLB | | 1.2378068 | NM_170662 | Cas-Br-M (murine) ecotropic retroviral transforming sequence b |
| PIK3CD | | 1.2172623 | NM_005026 | phosphoinositide-3-kinase, catalytic, delta polypeptide |
| EPS8 | | 1.205122 | NM_004447 | epidermal growth factor receptor pathway substrate 8 |
| SOS2 | | 1.0677557 | NM_006939 | son of sevenless homolog 2 (Drosophila) |
| JUND | | 1.0222626 | NM_005354 | jun D proto-oncogene |
| PRKCB | | -6.210244 | NM_002738 | protein kinase C, beta |
| C10orf81 | | -2.9325328 | NM_024889 | chromosome 10 open reading frame 81 |
| SHD | | -2.0695524 | NM_020209 | Src homology 2 domain containing transforming protein D |
| AKT3 | | -2.0072546 | NM_181690 | v-akt murine thymoma viral oncogene homolog 3 |
| GAB1 | | -1.5936689 | NM_207123 | GRB2-associated binding protein 1 |
| NOSTRIN | | -1.0960379 | NM_052946 | nitric oxide synthase trafficker |
| MAPK10 | | -1.0286255 | NM_138980 | mitogen-activated protein kinase 10 |
| **Focal adhesion** | | | | |
| SHC4 | | 4.224395 | NM_203349 | SHC (Src homology 2 domain containing) family, member 4 |
| VEGFC | | 3.8598404 | NM_005429 | vascular endothelial growth factor C |
| ITGB7 | | 3.234314 | NM_000889 | integrin, beta 7 |
| LOC100289495 | | 2.6865034 | NR_040022 | uncharacterized LOC100289495 |
| ITGA2 | | 2.6736517 | NM_002203 | integrin, alpha 2 (CD49B, alpha 2 subunit of VLA-2 receptor) |
| CLMN | | 2.3493013 | NM_024734 | calmin (calponin-like, transmembrane) |
| LAMB2P1 | | 2.211647 | NR_004405 | laminin, beta 2 pseudogene 1 |
| LAMA4 | | 2.1986122 | NM_001105206 | laminin, alpha 4 |
| MYLK | | 2.1521683 | NM_053025 | myosin light chain kinase |
| ITGB4 | | 2.0468588 | NM_000213 | integrin, beta 4 |
| JUNB | | 1.9544878 | NM_002229 | jun B proto-oncogene |
| ITGA6 | | 1.9236512 | NM_000210 | integrin, alpha 6 |
| JUN | | 1.8334875 | NM_002228 | jun proto-oncogene |
| SH3YL1 | | 1.691999 | NM_015677 | SH3 domain containing, Ysc84-like 1 (S. cerevisiae) |
| C14orf49 | | 1.6756778 | NM_152592 | chromosome 14 open reading frame 49 |
| LAMC2 | | 1.5982022 | NM_018891 | laminin, gamma 2 |
| MCTP1 | | 1.5820065 | NM_024717 | multiple C2 domains, transmembrane 1 |
| VEGFA | | 1.5091877 | NM_001025366 | vascular endothelial growth factor A |
| FBLIM1 | | 1.452425 | NM_001024215 | filamin binding LIM protein 1 |
| CCND1 | | 1.3763199 | NM_053056 | cyclin D1 |
| CDC42EP2 | | 1.3375244 | NM_006779 | CDC42 effector protein (Rho GTPase binding) 2 |
| ITGA1 | | 1.2995157 | NM_181501 | integrin, alpha 1 |
| BIRC3 | | 1.2701454 | NM_001165 | baculoviral IAP repeat containing 3 |
| PIK3CD | | 1.2172623 | NM_005026 | phosphoinositide-3-kinase, catalytic, delta polypeptide |
| MYLK4 | | 1.1573462 | NM_001012418 | myosin light chain kinase family, member 4 |
| FLJ44511 | | 1.124855 | NR_033963 | uncharacterized LOC441307 |
| PDLIM5 | | 1.0715475 | NM_006457 | PDZ and LIM domain 5 |
| SOS2 | | 1.0677557 | NM_006939 | son of sevenless homolog 2 (Drosophila) |
| COL4A1 | | 1.0665026 | NM_001845 | collagen, type IV, alpha 1 |
| FLJ42627 | | 1.0478392 | NR_024492 | uncharacterized LOC645644 |
| PDGFA | | 1.0315065 | NM_033023 | platelet-derived growth factor alpha polypeptide |
| JUND | | 1.0222626 | NM_005354 | jun D proto-oncogene |
| PRKCB | | -6.210244 | NM_002738 | protein kinase C, beta |
| OTOG | | -3.211523 | XM_001717531 | otogelin |
| LOC100289038 | | -3.1103573 | XR_132906 | von Willebrand factor-like |
| VWF | | -2.6140704 | NM_000552 | von Willebrand factor |
| ACTL8 | | -2.6115284 | NM_030812 | actin-like 8 |
| VAV3 | | -2.5884 | NM_006113 | vav 3 guanine nucleotide exchange factor |
| MYL9 | | -2.3301706 | NM_181526 | myosin, light chain 9, regulatory |
| MYLK3 | | -2.285798 | NM_182493 | myosin light chain kinase 3 |
| ITGBL1 | | -2.2509308 | NM_004791 | integrin, beta-like 1 (with EGF-like repeat domains) |
| LDB3 | | -2.1921978 | NM_001171610 | LIM domain binding 3 |
| AKT3 | | -2.0072546 | NM_181690 | v-akt murine thymoma viral oncogene homolog 3 |
| CAV1 | | -1.8263359 | NM_001753 | caveolin 1, caveolae protein, 22kDa |
| CAV3 | | -1.644249 | NM_001234 | caveolin 3 |
| COL24A1 | | -1.5017715 | NM_152890 | collagen, type XXIV, alpha 1 |
| MICALCL | | -1.4798837 | NM_032867 | MICAL C-terminal like |
| CAV2 | | -1.4614305 | NM_001233 | caveolin 2 |
| CDC42BPG | | -1.4571857 | NM_017525 | CDC42 binding protein kinase gamma (DMPK-like) |
| TNS1 | | -1.3698735 | NM_022648 | tensin 1 |
| LAMA4 | | -1.3583341 | NM_001105206 | laminin, alpha 4 |
| PLB1 | | -1.218164 | NM_153021 | phospholipase B1 |
| PGF | | -1.1488256 | NM_002632 | placental growth factor |
| CDC42 | | -1.1461239 | NM_044472 | cell division cycle 42 (GTP binding protein, 25kDa) |
| MYLK2 | | -1.1345067 | NM_033118 | myosin light chain kinase 2 |
| TNS3 | | -1.1107836 | NM_022748 | tensin 3 |
| VWA2 | | -1.1014357 | NM_198496 | von Willebrand factor A domain containing 2 |
| NOSTRIN | | -1.0960379 | NM_052946 | nitric oxide synthase trafficker |
| CHADL | | -1.0455093 | NM_138481 | chondroadherin-like |
| MAPK10 | | -1.0286255 | NM_138980 | mitogen-activated protein kinase 10 |
| **Hypertrophic cardiomyopathy (HCM)** | | | | |
| IL6 | | 3.9022264 | NM_000600 | interleukin 6 (interferon, beta 2) |
| CACNG6 | | 3.672966 | NM_145814 | calcium channel, voltage-dependent, gamma subunit 6 |
| ITGB7 | | 3.234314 | NM_000889 | integrin, beta 7 |
| ITGA2 | | 2.6736517 | NM_002203 | integrin, alpha 2 (CD49B, alpha 2 subunit of VLA-2 receptor) |
| ITGB4 | | 2.0468588 | NM_000213 | integrin, beta 4 |
| IFLTD1 | | 2.0200586 | NM_001145729 | intermediate filament tail domain containing 1 |
| ITGA6 | | 1.9236512 | NM_000210 | integrin, alpha 6 |
| IL6ST | | 1.4187331 | NM_002184 | interleukin 6 signal transducer (gp130, oncostatin M receptor) |
| ITGA1 | | 1.2995157 | NM_181501 | integrin, alpha 1 |
| DMD | | 1.2630501 | NM_004021 | dystrophin |
| CACNG8 | | 1.0947104 | NM_031895 | calcium channel, voltage-dependent, gamma subunit 8 |
| OBSCN | | 1.0740819 | BC043916 | obscurin, cytoskeletal calmodulin and titin-interacting RhoGEF |
| RYR2 | | -4.693291 | NM_001035 | ryanodine receptor 2 (cardiac) |
| EDN2 | | -3.1589665 | NM_001956 | endothelin 2 |
| ACTL8 | | -2.6115284 | NM_030812 | actin-like 8 |
| CACNB2 | | -2.2987142 | NM_000724 | calcium channel, voltage-dependent, beta 2 subunit |
| ITGBL1 | | -2.2509308 | NM_004791 | integrin, beta-like 1 (with EGF-like repeat domains) |
| EDN1 | | -1.7481451 | NM_001955 | endothelin 1 |
| DES | | -1.014029 | NM_001927 | desmin |
| **Leukocyte transendothelial migration** | | | | |
| RAPGEF4 | | 3.218923 | NM_007023 | Rap guanine nucleotide exchange factor (GEF) 4 |
| LOC100289495 | | 2.6865034 | NR_040022 | uncharacterized LOC100289495 |
| CLMN | | 2.3493013 | NM_024734 | calmin (calponin-like, transmembrane) |
| TXK | | 2.3223662 | NM_003328 | TXK tyrosine kinase |
| SIPA1L2 | | 2.3134384 | NM_020808 | signal-induced proliferation-associated 1 like 2 |
| RAPGEF3 | | 2.3085666 | NM_006105 | Rap guanine nucleotide exchange factor (GEF) 3 |
| CLDN1 | | 2.043603 | NM_021101 | claudin 1 |
| ICAM1 | | 1.9410534 | NM_000201 | intercellular adhesion molecule 1 |
| CLDN14 | | 1.874815 | NM_144492 | claudin 14 |
| C14orf49 | | 1.6756778 | NM_152592 | chromosome 14 open reading frame 49 |
| MCTP1 | | 1.5820065 | NM_024717 | multiple C2 domains, transmembrane 1 |
| CDC42EP2 | | 1.3375244 | NM_006779 | CDC42 effector protein (Rho GTPase binding) 2 |
| PIK3CD | | 1.2172623 | NM_005026 | phosphoinositide-3-kinase, catalytic, delta polypeptide |
| PECAM1 | | 1.1474533 | NM_000442 | platelet/endothelial cell adhesion molecule |
| NCF2 | | 1.0901747 | NM_000433 | neutrophil cytosolic factor 2 |
| PDLIM5 | | 1.0715475 | NM_006457 | PDZ and LIM domain 5 |
| SIPA1L1 | | 1.039587 | NM_015556 | signal-induced proliferation-associated 1 like 1 |
| SH3PXD2B | | 1.0339966 | NM_001017995 | SH3 and PX domains 2B |
| PRKCB | | -6.210244 | NM_002738 | protein kinase C, beta |
| NOX4 | | -4.229009 | NM_001143836 | NADPH oxidase 4 |
| ACTL8 | | -2.6115284 | NM_030812 | actin-like 8 |
| VAV3 | | -2.5884 | NM_006113 | vav 3 guanine nucleotide exchange factor |
| CLDN22 | | -2.5571098 | NM_001111319 | claudin 22 |
| CTNNA2 | | -2.4047928 | NM_004389 | catenin (cadherin-associated protein), alpha 2 |
| MYL9 | | -2.3301706 | NM_181526 | myosin, light chain 9, regulatory |
| ITGBL1 | | -2.2509308 | NM_004791 | integrin, beta-like 1 (with EGF-like repeat domains) |
| LDB3 | | -2.1921978 | NM_001171610 | LIM domain binding 3 |
| CLDN24 | | -1.6983728 | NM_001185149 | claudin 24 |
| JAM3 | | -1.5630703 | NM_032801 | junctional adhesion molecule 3 |
| MICALCL | | -1.4798837 | NM_032867 | MICAL C-terminal like |
| CDC42BPG | | -1.4571857 | NM_017525 | CDC42 binding protein kinase gamma (DMPK-like) |
| SIPA1L3 | | -1.3543863 | NM_015073 | signal-induced proliferation-associated 1 like 3 |
| RAPGEFL1 | | -1.3440132 | NM_016339 | Rap guanine nucleotide exchange factor (GEF)-like 1 |
| RASSF5 | | -1.1462722 | NM_182663 | Ras association (RalGDS/AF-6) domain family member 5 |
| CDC42 | | -1.1461239 | NM_044472 | cell division cycle 42 (GTP binding protein, 25kDa) |
| JAM2 | | -1.0542111 | AK056079 | junctional adhesion molecule 2 |
| Pathways in cancer | | | | |
| MMP1 | | 9.23877 | NM_002421 | matrix metallopeptidase 1 (interstitial collagenase) |
| IL8 | | 8.2453165 | NM_000584 | interleukin 8 |
| IL6 | | 3.9022264 | NM_000600 | interleukin 6 (interferon, beta 2) |
| GLI2 | | 3.899661 | NM_005270 | GLI family zinc finger 2 |
| VEGFC | | 3.8598404 | NM_005429 | vascular endothelial growth factor C |
| FOSL1 | | 3.4074564 | NM_005438 | FOS-like antigen 1 |
| CDKN1A | | 2.8123603 | NM_078467 | cyclin-dependent kinase inhibitor 1A (p21, Cip1) |
| IKBKB | | 2.7367592 | AK309052 | inhibitor of kappa light polypeptide gene enhancer in B-cells |
| ITGA2 | | 2.6736517 | NM_002203 | integrin, alpha 2 (CD49B, alpha 2 subunit of VLA-2 receptor) |
| CDKN2B | | 2.4228024 | NM_004936 | cyclin-dependent kinase inhibitor 2B (p15, inhibits CDK4) |
| LAMB2P1 | | 2.211647 | NR_004405 | laminin, beta 2 pseudogene 1 |
| LAMA4 | | 2.1986122 | NM_001105206 | laminin, alpha 4 |
| SHB | | 2.060567 | NM_003028 | Src homology 2 domain containing adaptor protein B |
| FOS | | 2.0178242 | NM_005252 | FBJ murine osteosarcoma viral oncogene homolog |
| JUNB | | 1.9544878 | NM_002229 | jun B proto-oncogene |
| CDH13 | | 1.9504576 | NM_001257 | cadherin 13, H-cadherin (heart) |
| ITGA6 | | 1.9236512 | NM_000210 | integrin, alpha 6 |
| JUN | | 1.8334875 | NM_002228 | jun proto-oncogene |
| SH3YL1 | | 1.691999 | NM_015677 | SH3 domain containing, Ysc84-like 1 (S. cerevisiae) |
| LAMC2 | | 1.5982022 | NM_018891 | laminin, gamma 2 |
| MCTP1 | | 1.5820065 | NM_024717 | multiple C2 domains, transmembrane 1 |
| TGFA | | 1.5458126 | NM_003236 | transforming growth factor, alpha |
| VEGFA | | 1.5091877 | NM_001025366 | vascular endothelial growth factor A |
| NFKB2 | | 1.4605198 | NM_001077493 | nuclear factor of kappa light polypeptide gene enhancer in B-cells 2 |
| MLH1 | | 1.4571791 | NM_000249 | mutL homolog 1, colon cancer, nonpolyposis type 2 (E. coli) |
| FOSB | | 1.4260874 | NM_006732 | FBJ murine osteosarcoma viral oncogene homolog B |
| IL6ST | | 1.4187331 | NM_002184 | interleukin 6 signal transducer (gp130, oncostatin M receptor) |
| NKX3-1 | | 1.3917985 | NM_006167 | NK3 homeobox 1 |
| CCND1 | | 1.3763199 | NM_053056 | cyclin D1 |
| WNT5B | | 1.3509774 | NM_030775 | wingless-type MMTV integration site family, member 5B |
| CDC42EP2 | | 1.3375244 | NM_006779 | CDC42 effector protein (Rho GTPase binding) 2 |
| BIRC3 | | 1.2701454 | NM_001165 | baculoviral IAP repeat containing 3 |
| BMP2 | | 1.2659893 | NM_001200 | bone morphogenetic protein 2 |
| CDH15 | | 1.2633142 | NM_004933 | cadherin 15, type 1, M-cadherin (myotubule) |
| MMP14 | | 1.2412271 | NM_004995 | matrix metallopeptidase 14 (membrane-inserted) |
| CBLB | | 1.2378068 | NM_170662 | Cas-Br-M (murine) ecotropic retroviral transforming sequence b |
| TCF7L2 | | 1.2279196 | NM_030756 | transcription factor 7-like 2 (T-cell specific, HMG-box) |
| PIK3CD | | 1.2172623 | NM_005026 | phosphoinositide-3-kinase, catalytic, delta polypeptide |
| FZD4 | | 1.2079163 | NM_012193 | frizzled family receptor 4 |
| PPARG | | 1.2038794 | NM_138711 | peroxisome proliferator-activated receptor gamma |
| DAPK2 | | 1.1961813 | NM_014326 | death-associated protein kinase 2 |
| FLJ44511 | | 1.124855 | NR_033963 | uncharacterized LOC441307 |
| TPRA1 | | 1.0835466 | NM_001142646 | transmembrane protein, adipocyte asscociated 1 |
| SOS2 | | 1.0677557 | NM_006939 | son of sevenless homolog 2 (Drosophila) |
| COL4A1 | | 1.0665026 | NM_001845 | collagen, type IV, alpha 1 |
| TCF7L1 | | 1.0414724 | NM_031283 | transcription factor 7-like 1 (T-cell specific, HMG-box) |
| PDGFA | | 1.0315065 | NM_033023 | platelet-derived growth factor alpha polypeptide |
| JUND | | 1.0222626 | NM_005354 | jun D proto-oncogene |
| PRKCB | | -6.210244 | NM_002738 | protein kinase C, beta |
| FGFR2 | | -5.07627 | NM_022970 | fibroblast growth factor receptor 2 |
| CDH19 | | -4.5434165 | NM_021153 | cadherin 19, type 2 |
| AXIN2 | | -4.330981 | NM_004655 | axin 2 |
| CDH10 | | -2.9090514 | NM_006727 | cadherin 10, type 2 (T2-cadherin) |
| FGF13 | | -2.8540053 | NM_004114 | fibroblast growth factor 13 |
| HSP90AA5P | | -2.7146144 | AY956761 | heat shock protein 90kDa alpha (cytosolic), class A member 5 |
| CDH18 | | -2.68962 | NM_004934 | cadherin 18, type 2 |
| CTNNA2 | | -2.4047928 | NM_004389 | catenin (cadherin-associated protein), alpha 2 |
| AKT3 | | -2.0072546 | NM_181690 | v-akt murine thymoma viral oncogene homolog 3 |
| MITF | | -1.8201065 | NM_198159 | microphthalmia-associated transcription factor |
| CDC42BPG | | -1.4571857 | NM_017525 | CDC42 binding protein kinase gamma (DMPK-like) |
| TNS1 | | -1.3698735 | NM_022648 | tensin 1 |
| LAMA4 | | -1.3583341 | NM_001105206 | laminin, alpha 4 |
| PGF | | -1.1488256 | NM_002632 | placental growth factor |
| RASSF5 | | -1.1462722 | NM_182663 | Ras association (RalGDS/AF-6) domain family member 5 |
| CDC42 | | -1.1461239 | NM_044472 | cell division cycle 42 (GTP binding protein, 25kDa) |
| STARD13 | | -1.138731 | NM_178006 | StAR-related lipid transfer (START) domain containing 13 |
| TNS3 | | -1.1107836 | NM_022748 | tensin 3 |
| NOSTRIN | | -1.0960379 | NM_052946 | nitric oxide synthase trafficker |
| TPR | | -1.068521 | NM_003292 | translocated promoter region (to activated MET oncogene) |
| CDH12 | | -1.0432005 | NM_004061 | cadherin 12, type 2 (N-cadherin 2) |
| MAPK10 | | -1.0286255 | NM_138980 | mitogen-activated protein kinase 10 |
| **Prostate cancer** | | | | |
| CDKN1A | | 2.8123603 | NM_078467 | cyclin-dependent kinase inhibitor 1A (p21, Cip1) |
| IKBKB | | 2.7367592 | AK309052 | inhibitor of kappa light polypeptide gene enhancer in B-cells |
| CREB5 | | 2.7145996 | NM_182898 | cAMP responsive element binding protein 5 |
| TGFA | | 1.5458126 | NM_003236 | transforming growth factor, alpha |
| NKX3-1 | | 1.3917985 | NM_006167 | NK3 homeobox 1 |
| CCND1 | | 1.3763199 | NM_053056 | cyclin D1 |
| TCF7L2 | | 1.2279196 | NM_030756 | transcription factor 7-like 2 (T-cell specific, HMG-box) |
| PIK3CD | | 1.2172623 | NM_005026 | phosphoinositide-3-kinase, catalytic, delta polypeptide |
| SOS2 | | 1.0677557 | NM_006939 | son of sevenless homolog 2 (Drosophila) |
| TCF7L1 | | 1.0414724 | NM_031283 | transcription factor 7-like 1 (T-cell specific, HMG-box) |
| FGFR2 | | -5.07627 | NM_022970 | fibroblast growth factor receptor 2 |
| AKT3 | | -2.0072546 | NM_181690 | v-akt murine thymoma viral oncogene homolog 3 |
| TNS3 | | -1.1107836 | NM_022748 | tensin 3 |
| NOSTRIN | | -1.0960379 | NM_052946 | nitric oxide synthase trafficker |
| **Tight junction** | | | | |
| MYH15 | | 2.978115 | NM_014981 | myosin, heavy chain 15 |
| CLMN | | 2.3493013 | NM_024734 | calmin (calponin-like, transmembrane) |
| EPB41L5 | | 2.0806236 | NM_020909 | erythrocyte membrane protein band 4.1 like 5 |
| CLDN1 | | 2.043603 | NM_021101 | claudin 1 |
| CLDN14 | | 1.874815 | NM_144492 | claudin 14 |
| C14orf49 | | 1.6756778 | NM_152592 | chromosome 14 open reading frame 49 |
| FRMD3 | | 1.634675 | NM_001244959 | FERM domain containing 3 |
| FAM138E | | 1.6081686 | NR_026819 | family with sequence similarity 138, member E |
| MYH11 | | 1.5923076 | NM_001040113 | myosin, heavy chain 11, smooth muscle |
| MCTP1 | | 1.5820065 | NM_024717 | multiple C2 domains, transmembrane 1 |
| PDLIM7 | | 1.4755363 | NM_005451 | PDZ and LIM domain 7 (enigma) |
| DLG5 | | 1.3655882 | NM_004747 | discs, large homolog 5 (Drosophila) |
| TJP2 | | 1.3627663 | NM_201629 | tight junction protein 2 (zona occludens 2) |
| CDC42EP2 | | 1.3375244 | NM_006779 | CDC42 effector protein (Rho GTPase binding) 2 |
| BEGAIN | | 1.3090181 | NM_001159531 | brain-enriched guanylate kinase-associated homolog (rat) |
| PRKCE | | 1.3001909 |  | protein kinase C, epsilon |
| PPP2R2D | | 1.0732155 | AB040974 | protein phosphatase 2, regulatory subunit B, delta |
| PDE4DIP | | 1.0408382 | NM_014644 | phosphodiesterase 4D interacting protein |
| PRKCB | | -6.210244 | NM_002738 | protein kinase C, beta |
| CGN | | -3.7738194 | NM_020770 | cingulin |
| ACTL8 | | -2.6115284 | NM_030812 | actin-like 8 |
| CLDN22 | | -2.5571098 | NM_001111319 | claudin 22 |
| CTNNA2 | | -2.4047928 | NM_004389 | catenin (cadherin-associated protein), alpha 2 |
| MYL9 | | -2.3301706 | NM_181526 | myosin, light chain 9, regulatory |
| PARD3B | | -2.1636863 | NM_152526 | par-3 partitioning defective 3 homolog B (C. elegans) |
| AKT3 | | -2.0072546 | NM_181690 | v-akt murine thymoma viral oncogene homolog 3 |
| MPP4 | | -1.7777424 | NM_033066 | membrane protein, palmitoylated 4 |
| CLDN24 | | -1.6983728 | NM_001185149 | claudin 24 |
| JAM3 | | -1.5630703 | NM_032801 | junctional adhesion molecule 3 |
| MAGIX | | -1.549037 | DQ884401 | MAGI family member, X-linked |
| MICALCL | | -1.4798837 | NM_032867 | MICAL C-terminal like |
| CDC42BPG | | -1.4571857 | NM_017525 | CDC42 binding protein kinase gamma (DMPK-like) |
| USH1C | | -1.4460344 | NM_005709 | Usher syndrome 1C (autosomal recessive, severe) |
| TNS1 | | -1.3698735 | NM_022648 | tensin 1 |
| VAPA | | -1.2228136 | NM_003574 | VAMP (vesicle-associated membrane protein)-associated protein A |
| MPP2 | | -1.1474643 | NM_005374 | membrane protein, palmitoylated 2 (MAGUK p55 subfamily member 2) |
| CDC42 | | -1.1461239 | NM_044472 | cell division cycle 42 (GTP binding protein, 25kDa) |
| TNS3 | | -1.1107836 | NM_022748 | tensin 3 |
| TJP3 | | -1.078188 | NM_014428 | tight junction protein 3 (zona occludens 3) |
| LLGL2 | | -1.0565939 | NM_001015002 | lethal giant larvae homolog 2 (Drosophila) |
| JAM2 | | -1.0542111 | AK056079 | junctional adhesion molecule 2 |
| EPB41 | | -1.0072398 | NM_203342 | erythrocyte membrane protein band 4.1 (elliptocytosis 1, RH-linked) |

All these pathway: P<0.05
