# Supplementary material for: Native musk and synthetic musk ketone strongly induced the growth repression and the apoptosis of cancer cells
Source: BMC Complement Altern Med. 2016 Dec 8;16:511. doi: 10.1186/s12906-016-1493-2 (PMC5146870; doi:10.1186/s12906-016-1493-2)
Supplement: Additional file 4: — Differentially expressed genes in XL-JT after treatment with native musk. (DOC 655 kb) [file 12906_2016_1493_MOESM4_ESM.doc]

**Additional file 4.** Differentially expressed genes in XL-JT after native musk treatment.

| **GeneSymbol** | **Log2( Fold change)** | **Genbank** | **Gene titile** |
| --- | --- | --- | --- |
| **Cytokine-cytokine receptor interaction** | | | |
| **IL family** |  |  |  |
| IL24 | 8.044937 | NM_001185156 | interleukin 24 |
| IL20 | 2.7862678 | NM_018724 | interleukin 20 |
| IL1B | 2.6112733 | NM_000576 | interleukin 1, beta |
| IL6 | 2.5217304 | AK298077 | interleukin 6 (interferon, beta 2) |
| IL21R | 2.2348156 | NM_181078 | interleukin 21 receptor |
| IL15RA | 2.1095634 | NM_172200 | interleukin 15 receptor, alpha |
| IL8 | 2.0874815 | NM_000584 | interleukin 8 |
| IL28A | 2.0185246 | NM_172138 | interleukin 28A (interferon, lambda 2) |
| IL31RA | 1.7737079 | NM_001242638 | interleukin 31 receptor A |
| IL1A | 1.5160174 | NM_000575 | interleukin 1, alpha |
| IL7 | 1.2808261 | NM_000880 | interleukin 7 |
| IL28RA | 1.2088976 | NM_170743 | interleukin 28 receptor, alpha (interferon, lambda receptor) |
| IL10RB | 1.1823816 | NM_000628 | interleukin 10 receptor, beta |
| IL23A | 1.1551957 | NM_016584 | interleukin 23, alpha subunit p19 |
| IL6R | 1.0658541 | NM_000565 | interleukin 6 receptor |
| IL5RA | -6.1708956 | NM_175725 | interleukin 5 receptor, alpha |
| IL10RA | -3.948368 | NM_001558 | interleukin 10 receptor, alpha |
| IL1R2 | -3.4194913 | NM_004633 | interleukin 1 receptor, type II |
| IL11 | -1.4855204 | NM_000641 | interleukin 11 |
| IL1R1 | -1.1269131 | NM_000877 | interleukin 1 receptor, type I |
| **TNF family** |  |  |  |
| TNFRSF1B | 1.7558923 | NM_001066 | tumor necrosis factor receptor superfamily, member 1B |
| TNFRSF25 | 1.7208767 | NM_148965 | tumor necrosis factor receptor superfamily, member 25 |
| TNFSF4 | 1.3073354 | NM_003326 | tumor necrosis factor (ligand) superfamily, member 4 |
| TNFSF11 | -5.1105146 | NM_033012 | tumor necrosis factor (ligand) superfamily, member 11 |
| TNFSF15 | -3.339375 | NM_005118 | tumor necrosis factor (ligand) superfamily, member 15 |
| TNFSF10 | -1.0456581 | NM_003810 | tumor necrosis factor (ligand) superfamily, member 10 |
| **Other families** |  |  |  |
| NGFR | 4.5845394 | NM_002507 | nerve growth factor receptor |
| CXCR4 | 2.7701635 | NM_001008540 | chemokine (C-X-C motif) receptor 4 |
| CSF2 | 2.1559277 | NM_000758 | colony stimulating factor 2 (granulocyte-macrophage) |
| VEGFA | 2.123829 | NM_001025366 | vascular endothelial growth factor A |
| ACVR1C | 2.0870385 | NM_145259 | activin A receptor, type IC |
| OSM | 1.9315605 | NM_020530 | oncostatin M |
| BMP2 | 1.920506 | NM_001200 | bone morphogenetic protein 2 |
| INHBE | 1.7669902 | NM_031479 | inhibin, beta E |
| OSMR | 1.7326956 | NM_001168355 | oncostatin M receptor |
| INHBA | 1.6036229 | NM_002192 | inhibin, beta A |
| EDA2R | 1.5590158 | NM_021783 | ectodysplasin A2 receptor |
| CCL20 | 1.4581709 | NM_004591 | chemokine (C-C motif) ligand 20 |
| CSF2RA | 1.4420996 | NM_172249 | colony stimulating factor 2 receptor, alpha |
| PGF | 1.4288907 | NM_002632 | placental growth factor |
| PLEKHG5 | 1.2904925 | NM_198681 | pleckstrin homology domain containing, family G |
| ACVR1 | 1.2251892 | NM_001105 | activin A receptor, type I |
| RUFY4 | 1.1043453 | NM_198483 | RUN and FYVE domain containing 4 |
| FLJ44511 | 1.0817823 | NR_033963 | uncharacterized LOC441307 |
| BMP5 | -3.0979009 | NM_021073 | bone morphogenetic protein 5 |
| PDGFRA | -3.0847974 | NM_006206 | platelet-derived growth factor receptor, alpha polypeptide |
| MATN2 | -2.3641481 | NM_030583 | matrilin 2 |
| HGF | -2.3283253 | NM_001010934 | hepatocyte growth factor (hepapoietin A; scatter factor) |
| HGF | -2.0814676 | NM_001010931 | hepatocyte growth factor (hepapoietin A; scatter factor) |
| EDA | -2.001011 | NM_001399 | ectodysplasin A |
| CCL2 | -1.9481001 | NM_002982 | chemokine (C-C motif) ligand 2 |
| PDGFD | -1.8911943 | NM_025208 | platelet derived growth factor D |
| CCR7 | -1.8799787 | NM_001838 | chemokine (C-C motif) receptor 7 |
| CSF1 | -1.8144355 | NM_172210 | colony stimulating factor 1 (macrophage) |
| LTB | -1.6449728 | NM_002341 | lymphotoxin beta (TNF superfamily, member 3) |
| CXCR7 | -1.3908157 | NM_020311 | chemokine (C-X-C motif) receptor 7 |
| INHBB | -1.3709555 | NM_002193 | inhibin, beta B |
| BMP4 | -1.3264532 | NM_001202 | bone morphogenetic protein 4 |
| CXCL1 | -1.2118177 | NM_001511 | chemokine (C-X-C motif) ligand 1 |
| TNS4 | -1.0627546 | NM_032865 | tensin 4 |
| BMPR1B | -1.047821 | NM_001203 | bone morphogenetic protein receptor, type IB |
| CLCF1 | -1.0301905 | NM_013246 | cardiotrophin-like cytokine factor 1 |
| Jak-STAT signaling pathway | |  |  |
| IL24 | 8.044937 | NM_001185156 | interleukin 24 |
| CCND2 | 3.5426855 | NM_001759 | cyclin D2 |
| IL20 | 2.7862678 | NM_018724 | interleukin 20 |
| IL6 | 2.5217304 | AK298077 | interleukin 6 (interferon, beta 2) |
| IL21R | 2.2348156 | NM_181078 | interleukin 21 receptor |
| CSF2 | 2.1559277 | NM_000758 | colony stimulating factor 2 (granulocyte-macrophage) |
| IL15RA | 2.1095634 | NM_172200 | interleukin 15 receptor, alpha |
| IL28A | 2.0185246 | NM_172138 | interleukin 28A (interferon, lambda 2) |
| OSM | 1.9315605 | NM_020530 | oncostatin M |
| IL31RA | 1.7737079 | NM_001242638 | interleukin 31 receptor A |
| OSMR | 1.7326956 | NM_001168355 | oncostatin M receptor |
| SH3YL1 | 1.4931707 | NM_015677 | SH3 domain containing, Ysc84-like 1 (S. cerevisiae) |
| PIK3CD | 1.4696407 | NM_005026 | phosphoinositide-3-kinase, catalytic, delta polypeptide |
| SOCS1 | 1.4626794 | NM_003745 | suppressor of cytokine signaling 1 |
| CSF2RA | 1.4420996 | NM_172249 | colony stimulating factor 2 receptor, alpha |
| CEP170 | 1.3645916 | NM_014812 | centrosomal protein 170kDa |
| IL7 | 1.2808261 | NM_000880 | interleukin 7 |
| IL28RA | 1.2088976 | NM_170743 | interleukin 28 receptor, alpha (interferon, lambda receptor) |
| IL10RB | 1.1823816 | NM_000628 | interleukin 10 receptor, beta |
| IL23A | 1.1551957 | NM_016584 | interleukin 23, alpha subunit p19 |
| SPRY1 | 1.1457367 | NM_199327 | sprouty homolog 1, antagonist of FGF signaling (Drosophila) |
| CBLB | 1.1337862 | NM_170662 | Cas-Br-M (murine) |
| IL6R | 1.0658541 | NM_000565 | interleukin 6 receptor |
| JAK3 | 1.0506487 | NM_000215 | Janus kinase 3 |
| CCND3 | -2.4006195 | NM_001760 | cyclin D3 |
| IL11 | -1.4855204 | NM_000641 | interleukin 11 |
| MAP7D3 | -1.0685081 | NM_001173517 | MAP7 domain containing 3 |
| CLCF1 | -1.0301905 | NM_013246 | cardiotrophin-like cytokine factor 1 |
| **p53 signaling pathway** | | | |
| CCND2 | 3.5426855 | NM_001759 | cyclin D2 |
| IGFBP1 | 3.151269 | NM_000596 | insulin-like growth factor binding protein 1 |
| SESN2 | 2.592866 | NM_031459 | sestrin 2 |
| CCNG2 | 2.350357 | NM_004354 | cyclin G2 |
| CNTD2 | 2.126494 | NM_024877 | cyclin N-terminal domain containing 2 |
| MDM2 | 1.8906732 | NM_002392 | Mdm2 p53 binding protein homolog (mouse) |
| GADD45A | 1.4208784 | NM_001924 | growth arrest and DNA-damage-inducible, alpha |
| ZMAT3 | 1.3364906 | NM_022470 | zinc finger, matrin-type 3 |
| BBC3 | 1.3061085 | NM_014417 | BCL2 binding component 3 |
| MDM4 | 1.2971373 | NM_002393 | Mdm4 p53 binding protein homolog (mouse) |
| CDK13 | 1.2584047 | NM_031267 | cyclin-dependent kinase 13 |
| BAI1 | 1.1821928 | NM_001702 | brain-specific angiogenesis inhibitor 1 |
| GADD45G | 1.1424665 | NM_006705 | growth arrest and DNA-damage-inducible, gamma |
| CDK1 | -2.5203757 | NM_001170406 | cyclin-dependent kinase 1 |
| IGFBP5 | -2.5032835 | NM_000599 | insulin-like growth factor binding protein 5 |
| RRM2 | -2.4229622 | NM_001034 | ribonucleotide reductase M2 |
| CCND3 | -2.4006195 | NM_001760 | cyclin D3 |
| CCNE2 | -2.351235 | NM_057749 | cyclin E2 |
| TNS1 | -2.2279205 | NM_022648 | tensin 1 |
| UPK1B | -1.7433662 | NM_006952 | uroplakin 1B |
| CCNB1 | -1.6495781 | NM_031966 | cyclin B1 |
| CDK2 | -1.1768713 | NM_001798 | cyclin-dependent kinase 2 |
| CCNB2 | -1.1293583 | NM_004701 | cyclin B2 |
| TSPAN18 | -1.0397491 | NM_130783 | tetraspanin 18 |
| **MAPK signaling pathway** | |  |  |
| RPS6KA2 | 5.58799 | NM_021135 | ribosomal protein S6 kinase, 90kDa, polypeptide 2 |
| PTPRR | 5.587762 | NM_002849 | protein tyrosine phosphatase, receptor type, R |
| NGFR | 4.5845394 | NM_002507 | nerve growth factor receptor |
| STMN4 | 3.9355316 | NM_030795 | stathmin-like 4 |
| DDIT3 | 3.063755 | NM_004083 | DNA-damage-inducible transcript 3 |
| IL1B | 2.6112733 | NM_000576 | interleukin 1, beta |
| FOSB | 2.611113 | NM_006732 | FBJ murine osteosarcoma viral oncogene homolog B |
| FGFR2 | 2.4704294 | NM_022970 | fibroblast growth factor receptor 2 |
| FGF21 | 2.3099008 | NM_019113 | fibroblast growth factor 21 |
| DUSP5 | 2.2149982 | NM_004419 | dual specificity phosphatase 5 |
| ICA1 | 2.1959887 | NM_004968 | islet cell autoantigen 1, 69kDa |
| STK32A | 2.1454034 | NM_145001 | serine/threonine kinase 32A |
| BAIAP3 | 1.9779124 | NM_003933 | BAI1-associated protein 3 |
| FGF8 | 1.941227 | NM_033163 | fibroblast growth factor 8 (androgen-induced) |
| CACNA1A | 1.8636465 | NM_023035 | calcium channel, voltage-dependent, P/Q type, alpha 1A subunit |
| TAB2 | 1.8235292 | NM_015093 | TGF-beta activated kinase 1/MAP3K7 binding protein 2 |
| ERAS | 1.8069363 | NM_181532 | ES cell expressed Ras |
| MCTP1 | 1.7965755 | NM_024717 | multiple C2 domains, transmembrane 1 |
| FGF11 | 1.7769985 | NM_004112 | fibroblast growth factor 11 |
| NGF | 1.6013422 | NM_002506 | nerve growth factor (beta polypeptide) |
| IL1A | 1.5160174 | NM_000575 | interleukin 1, alpha |
| SH3YL1 | 1.4931707 | NM_015677 | SH3 domain containing, Ysc84-like 1 (S. cerevisiae) |
| FGF18 | 1.4491901 | NM_003862 | fibroblast growth factor 18 |
| PLA2G4C | 1.4468822 | NM_003706 | phospholipase A2, group IVC (cytosolic, calcium-independent) |
| MAP3K5 | 1.4225483 | NM_005923 | mitogen-activated protein kinase kinase kinase 5 |
| GADD45A | 1.4208784 | NM_001924 | growth arrest and DNA-damage-inducible, alpha |
| RASAL2 | 1.4197006 | NM_170692 | RAS protein activator like 2 |
| FGFR3 | 1.3849258 | NM_000142 | fibroblast growth factor receptor 3 |
| MEF2C | 1.3678322 | NM_002397 | myocyte enhancer factor 2C |
| CEP170 | 1.3645916 | NM_014812 | centrosomal protein 170kDa |
| DUSP8 | 1.2907124 | NM_004420 | dual specificity phosphatase 8 |
| MAP4K3 | 1.2745314 | NM_003618 | mitogen-activated protein kinase kinase kinase kinase 3 |
| MAPK13 | 1.2583232 | NM_002754 | mitogen-activated protein kinase 13 |
| PRDM16 | 1.2317142 | NM_199454 | PR domain containing 16 |
| JUN | 1.2224321 | NM_002228 | jun proto-oncogene |
| FOSL1 | 1.191699 | NM_005438 | FOS-like antigen 1 |
| MAPK6 | 1.1907282 | NM_002748 | mitogen-activated protein kinase 6 |
| FOS | 1.1837597 | NM_005252 | FBJ murine osteosarcoma viral oncogene homolog |
| MAP3K2 | 1.1465826 | NM_006609 | mitogen-activated protein kinase kinase kinase 2 |
| GADD45G | 1.1424665 | NM_006705 | growth arrest and DNA-damage-inducible, gamma |
| DUSP16 | 1.1415424 | NM_030640 | dual specificity phosphatase 16 |
| DFNB31 | 1.1109138 | NM_015404 | deafness, autosomal recessive 31 |
| FLJ44511 | 1.0817823 | NR_033963 | uncharacterized LOC441307 |
| PLA2G2F | 1.0774126 | NM_022819 | phospholipase A2, group IIF |
| BDNF-AS1 | 1.0586333 | NR_002832 | BDNF antisense RNA 1 (non-protein coding) |
| RELA | 1.0390921 | NM_021975 | v-rel reticuloendotheliosis viral oncogene homolog A (avian) |
| ZC3H12C | 1.0256939 | NM_033390 | zinc finger CCCH-type containing 12C |
| ICA1L | -4.312938 | NM_178231 | islet cell autoantigen 1,69kDa-like |
| IL1R2 | -3.4194913 | NM_004633 | interleukin 1 receptor, type II |
| STK3 | -3.2547588 | AK075229 | serine/threonine kinase 3 |
| PDGFRA | -3.0847974 | NM_006206 | platelet-derived growth factor receptor, alpha polypeptide |
| CIB4 | -2.939083 | NM_001029881 | calcium and integrin binding family member 4 |
| MAP2K6 | -2.7985868 | NM_002758 | mitogen-activated protein kinase kinase 6 |
| CACNG4 | -2.4353113 | NM_014405 | calcium channel, voltage-dependent, gamma subunit 4 |
| ARRB1 | -2.4051275 | NM_004041 | arrestin, beta 1 |
| MATN2 | -2.3641481 | NM_030583 | matrilin 2 |
| SYNGR4 | -2.24798 | NM_012451 | synaptogyrin 4 |
| FGF22 | -2.2342987 | NM_020637 | fibroblast growth factor 22 |
| CD14 | -1.8981333 | NM_001174104 | CD14 molecule |
| MAPK9 | -1.6351929 | NM_001135044 | mitogen-activated protein kinase 9 |
| BDNF | -1.595644 | NM_170735 | brain-derived neurotrophic factor |
| MAP3K8 | -1.3073425 | NM_005204 | mitogen-activated protein kinase kinase kinase 8 |
| PLA2G1B | -1.2563686 | NM_000928 | phospholipase A2, group IB (pancreas) |
| MED11 | -1.2499142 | NM_001001683 | mediator complex subunit 11 |
| HSPA2 | -1.2199697 | NM_021979 | heat shock 70kDa protein 2 |
| IL1R1 | -1.1269131 | NM_000877 | interleukin 1 receptor, type I |
| CACNA1G | -1.1253948 | NM_018896 | calcium channel, voltage-dependent, T type, alpha 1G subunit |
| TESC | -1.0766335 | NM_017899 | tescalcin |
| YLPM1 | -1.0751219 | L40403 | YLP motif containing 1 |
| FGFR3 | -1.0739574 | NM_000142 | fibroblast growth factor receptor 3 |
| KSR2 | -1.0353942 | NM_173598 | kinase suppressor of ras 2 |
| PRKACA | -1.0325146 | NM_002730 | protein kinase, cAMP-dependent, catalytic, alpha |
| **Alanine, aspartate and glutamate metabolism** | | |  |
| GAD1 | 3.0496583 | NM_000817 | glutamate decarboxylase 1 (brain, 67kDa) |
| IFFO1 | 2.2956276 | NM_001039670 | intermediate filament family orphan 1 |
| FLJ43315 | 1.8176241 | NR_033856 | asparagine synthetase pseudogene |
| GFPT1 | 1.5583076 | NM_001244710 | glutamine--fructose-6-phosphate transaminase 1 |
| LOC442028 | 1.5556688 | NR_037597 | uncharacterized LOC442028 |
| ABAT | 1.3336592 | NM_000663 | 4-aminobutyrate aminotransferase |
| GPT2 | 1.2895889 | NM_133443 | glutamic pyruvate transaminase (alanine aminotransferase) 2 |
| ASS1 | 1.1728859 | NM_000050 | argininosuccinate synthase 1 |
| GLS | 1.0751734 | AF097492 | glutaminase |
| ALDH4A1 | -1.8116102 | NM_170726 | aldehyde dehydrogenase 4 family, member A1 |
| **Nicotinate and nicotinamide metabolism** | | |  |
| NMNAT2 | 1.8976831 | NM_015039 | nicotinamide nucleotide adenylyltransferase 2 |
| FAM3C | 1.5068617 | NM_014888 | family with sequence similarity 3, member C |
| KIAA1199 | 1.3620381 | NM_018689 | KIAA1199 |
| C9orf95 | 1.317339 | NM_017881 | chromosome 9 open reading frame 95 |
| NT5C1A | 1.0438786 | NM_032526 | 5'-nucleotidase, cytosolic IA |
| MFNG | 1.0249748 | NM_002405 | MFNG O-fucosylpeptide 3-beta-N-acetylglucosaminyltransferase |
| NT5C1B | -5.8073235 | NM_001002006 | 5'-nucleotidase, cytosolic IB |
| UGT2B15 | -4.265785 | NM_001076 | UDP glucuronosyltransferase 2 family, polypeptide B15 |
| UGT1A6 | -4.121839 | NM_001072 | UDP glucuronosyltransferase 1 family, polypeptide A6 |
| UGT1A8 | -3.737194 | NM_019076 | UDP glucuronosyltransferase 1 family, polypeptide A8 |
| FUT9 | -3.5549989 | NM_006581 | fucosyltransferase 9 (alpha (1,3) fucosyltransferase) |
| MGAT5B | -2.0065002 | NM_144677 | mannosyl |
| UGT2B11 | -1.7610216 | NM_001073 | UDP glucuronosyltransferase 2 family, polypeptide B11 |
| ST6GAL2 | -1.5792961 | NM_032528 | ST6 beta-galactosamide alpha-2,6-sialyltranferase 2 |
| LFNG | -1.4765387 | NM_001040167 | LFNG O-fucosylpeptide 3-beta-N-acetylglucosaminyltransferase |
| BST1 | -1.2988052 | NM_004334 | bone marrow stromal cell antigen 1 |
| ENPP1 | -1.1542635 | NM_006208 | ectonucleotide pyrophosphatase/phosphodiesterase 1 |
| NNMT | -1.0818834 | NM_006169 | nicotinamide N-methyltransferase |
| NT5DC4 | -1.0525346 | BC041437 | 5'-nucleotidase domain containing 4 |
| UGT2B7 | -1.0154257 | NM_001074 | UDP glucuronosyltransferase 2 family, polypeptide B7 |
| **Osteoclast differentiation** | | | |
| IL1B | 2.6112733 | NM_000576 | interleukin 1, beta |
| FOSB | 2.611113 | NM_006732 | FBJ murine osteosarcoma viral oncogene homolog B |
| GAB3 | 2.4396715 | NM_001081573 | GRB2-associated binding protein 3 |
| GAB2 | 2.2350893 | NM_012296 | GRB2-associated binding protein 2 |
| TAB2 | 1.8235292 | NM_015093 | TGF-beta activated kinase 1/MAP3K7 binding protein 2 |
| A1BG | 1.7994518 | NM_130786 | alpha-1-B glycoprotein |
| FCRLA | 1.6190362 | NM_032738 | Fc receptor-like A |
| LILRB3 | 1.605607 | NM_006864 | leukocyte immunoglobulin-like receptor, subfamily B |
| IL1A | 1.5160174 | NM_000575 | interleukin 1, alpha |
| SH3YL1 | 1.4931707 | NM_015677 | SH3 domain containing, Ysc84-like 1 (S. cerevisiae) |
| PIK3CD | 1.4696407 | NM_005026 | phosphoinositide-3-kinase, catalytic, delta polypeptide |
| SOCS1 | 1.4626794 | NM_003745 | suppressor of cytokine signaling 1 |
| CEP170 | 1.3645916 | NM_014812 | centrosomal protein 170kDa |
| MAPK13 | 1.2583232 | NM_002754 | mitogen-activated protein kinase 13 |
| SIRPD | 1.2356806 | NM_178460 | signal-regulatory protein delta |
| JUN | 1.2224321 | NM_002228 | jun proto-oncogene |
| FOSL1 | 1.191699 | NM_005438 | FOS-like antigen 1 |
| FOS | 1.1837597 | NM_005252 | FBJ murine osteosarcoma viral oncogene homolog |
| CTSK | 1.0932789 | NM_000396 | cathepsin K |
| RELA | 1.0390921 | NM_021975 | v-rel reticuloendotheliosis viral oncogene homolog A (avian) |
| LILRA5 | -5.227747 | NM_181879 | leukocyte immunoglobulin-like receptor, subfamily A |
| TNFSF11 | -5.1105146 | NM_033012 | tumor necrosis factor (ligand) superfamily, member 11 |
| NOX4 | -4.105172 | NM_001143836 | NADPH oxidase 4 |
| FCGR2A | -3.1883326 | NM_001136219 | Fc fragment of IgG, low affinity IIa, receptor (CD32) |
| MAP2K6 | -2.7985868 | NM_002758 | mitogen-activated protein kinase kinase 6 |
| SYNGR4 | -2.24798 | NM_012451 | synaptogyrin 4 |
| IGSF10 | -1.9277773 | NM_178822 | immunoglobulin superfamily, member 10 |
| CSF1 | -1.8144355 | NM_172210 | colony stimulating factor 1 (macrophage) |
| SIRPG | -1.645608 | NM_001039508 | signal-regulatory protein gamma |
| MAPK9 | -1.6351929 | NM_001135044 | mitogen-activated protein kinase 9 |
| IGSF1 | -1.5217705 | NM_205833 | immunoglobulin superfamily, member 1 |
| SIRPB2 | -1.4402118 | NM_001122962 | signal-regulatory protein beta 2 |
| VSTM1 | -1.3153868 | NM_198481 | V-set and transmembrane domain containing 1 |
| SIRPB1 | -1.3003411 | NM_001135844 | signal-regulatory protein beta 1 |
| IL1R1 | -1.1269131 | NM_000877 | interleukin 1 receptor, type I |
| SYNGR1 | -1.0944843 | NM_145738 | synaptogyrin 1 |
| YLPM1 | -1.0751219 | L40403 | YLP motif containing 1 |
| MAP7D3 | -1.0685081 | NM_001173517 | MAP7 domain containing 3 |
| **Retinol metabolism** | | | |
| RDH8 | 2.1729522 | NM_015725 | retinol dehydrogenase 8 (all-trans) |
| CYP1A1 | 1.99156 | NM_000499 | cytochrome P450, family 1, subfamily A, polypeptide 1 |
| DHRS9 | 1.8241363 | NM_005771 | dehydrogenase/reductase (SDR family) member 9 |
| SDR16C5 | 1.406959 | NM_138969 | short chain dehydrogenase/reductase family 16C, member 5 |
| UGT2B15 | -4.265785 | NM_001076 | UDP glucuronosyltransferase 2 family, polypeptide B15 |
| UGT1A6 | -4.121839 | NM_001072 | UDP glucuronosyltransferase 1 family, polypeptide A6 |
| CYP4A11 | -3.8628726 | NM_000778 | cytochrome P450, family 4, subfamily A, polypeptide 11 |
| UGT1A8 | -3.737194 | NM_019076 | UDP glucuronosyltransferase 1 family, polypeptide A8 |
| BCMO1 | -3.4024582 | NM_017429 | beta-carotene 15,15'-monooxygenase 1 |
| CYP26A1 | -2.5820856 | NM_057157 | cytochrome P450, family 26, subfamily A, polypeptide 1 |
| ALDH1A1 | -1.8248768 | NM_000689 | aldehyde dehydrogenase 1 family, member A1 |
| UGT2B11 | -1.7610216 | NM_001073 | UDP glucuronosyltransferase 2 family, polypeptide B11 |
| HRASLS2 | -1.075386 | NM_017878 | HRAS-like suppressor 2 |
| UGT2B7 | -1.0154257 | NM_001074 | UDP glucuronosyltransferase 2 family, polypeptide B7 |
| **Rheumatoid arthritis** | |  |  |
| ATP6V0D2 | 6.4942374 | NM_152565 | ATPase, H+ transporting, lysosomal 38kDa, V0 subunit d2 |
| MMP1 | 6.377101 | NM_002421 | matrix metallopeptidase 1 (interstitial collagenase) |
| MMP10 | 5.076376 | NM_002425 | matrix metallopeptidase 10 (stromelysin 2) |
| IL1B | 2.6112733 | NM_000576 | interleukin 1, beta |
| FOSB | 2.611113 | NM_006732 | FBJ murine osteosarcoma viral oncogene homolog B |
| IL6 | 2.5217304 | AK298077 | interleukin 6 (interferon, beta 2) |
| CSF2 | 2.1559277 | NM_000758 | colony stimulating factor 2 (granulocyte-macrophage) |
| VEGFA | 2.123829 | NM_001025366 | vascular endothelial growth factor A |
| IL8 | 2.0874815 | NM_000584 | interleukin 8 |
| CTSL1 | 1.7763214 |  | cathepsin L1 |
| ATP6AP1L | 1.5307455 | NM_001017971 | ATPase, H+ transporting, lysosomal accessory protein 1-like |
| IL1A | 1.5160174 | NM_000575 | interleukin 1, alpha |
| CSF2RA | 1.4420996 | NM_172249 | colony stimulating factor 2 receptor, alpha |
| PGF | 1.4288907 | NM_002632 | placental growth factor |
| LOC497256 | 1.2594938 | AK094988 | uncharacterized LOC497256 |
| MMP14 | 1.2578588 | NM_004995 | matrix metallopeptidase 14 (membrane-inserted) |
| JUN | 1.2224321 | NM_002228 | jun proto-oncogene |
| FOSL1 | 1.191699 | NM_005438 | FOS-like antigen 1 |
| FOS | 1.1837597 | NM_005252 | FBJ murine osteosarcoma viral oncogene homolog |
| CTSL1P2 | 1.182435 | NR_033407 | cathepsin L1 pseudogene 2 |
| IL23A | 1.1551957 | NM_016584 | interleukin 23, alpha subunit p19 |
| CTSK | 1.0932789 | NM_000396 | cathepsin K |
| IL6R | 1.0658541 | NM_000565 | interleukin 6 receptor |
| TNFSF11 | -5.1105146 | NM_033012 | tumor necrosis factor (ligand) superfamily, member 11 |
| ITGAL | -2.9365792 | NM_002209 | integrin, alpha L |
| HLA-DMB | -2.1501436 | NM_002118 | major histocompatibility complex, class II, DM beta |
| CSF1 | -1.8144355 | NM_172210 | colony stimulating factor 1 (macrophage) |
| LTB | -1.6449728 | NM_002341 | lymphotoxin beta (TNF superfamily, member 3) |
| IL11 | -1.4855204 | NM_000641 | interleukin 11 |
| HLA-DMA | -1.3124399 | NM_006120 | major histocompatibility complex, class II, DM alpha |
| **Steroid hormone biosynthesis** | | | |
| HSD17B3 | 3.2554407 | NM_000197 | hydroxysteroid (17-beta) dehydrogenase 3 |
| HSD17B7 | 3.0240784 | AK022929 | hydroxysteroid (17-beta) dehydrogenase 7 |
| CYP21A2 | 2.61339 | NM_000500 | cytochrome P450, family 21, subfamily A, polypeptide 2 |
| CYP1A1 | 1.99156 | NM_000499 | cytochrome P450, family 1, subfamily A, polypeptide 1 |
| SRD5A2 | 1.1664386 | NM_000348 | steroid-5-alpha-reductase, alpha polypeptide 2 |
| HSD17B14 | 1.1490679 | NM_016246 | hydroxysteroid (17-beta) dehydrogenase 14 |
| UGT2B15 | -4.265785 | NM_001076 | UDP glucuronosyltransferase 2 family, polypeptide B15 |
| UGT1A6 | -4.121839 | NM_001072 | UDP glucuronosyltransferase 1 family, polypeptide A6 |
| UGT1A8 | -3.737194 | NM_019076 | UDP glucuronosyltransferase 1 family, polypeptide A8 |
| SULT2B1 | -1.8910284 | NM_004605 | sulfotransferase family, cytosolic, 2B, member 1 |
| UGT2B11 | -1.7610216 | NM_001073 | UDP glucuronosyltransferase 2 family, polypeptide B11 |
| HSD17B6 | -1.3869944 | NM_003725 | hydroxysteroid (17-beta) dehydrogenase 6 homolog (mouse) |
| UGT2B7 | -1.0154257 | NM_001074 | UDP glucuronosyltransferase 2 family, polypeptide B7 |
| **Ascorbate and aldarate metabolism** | | | |
| MIOX | 4.1218514 | NM_017584 | myo-inositol oxygenase |
| ALDH8A1 | 1.4613128 | NM_022568 | aldehyde dehydrogenase 8 family, member A1 |
| UGT2B15 | -4.265785 | NM_001076 | UDP glucuronosyltransferase 2 family, polypeptide B15 |
| UGT1A6 | -4.121839 | NM_001072 | UDP glucuronosyltransferase 1 family, polypeptide A6 |
| UGT1A8 | -3.737194 | NM_019076 | UDP glucuronosyltransferase 1 family, polypeptide A8 |
| UGT2B11 | -1.7610216 | NM_001073 | UDP glucuronosyltransferase 2 family, polypeptide B11 |
| ALDH7A1 | -1.1198368 | NM_001182 | aldehyde dehydrogenase 7 family, member A1 |
| UGT2B7 | -1.0154257 | NM_001074 | UDP glucuronosyltransferase 2 family, polypeptide B7 |
| **Butirosin and neomycin biosynthesis** | | | |
| HK2 | 3.4536276 | NM_000189 | hexokinase 2 |
| HKDC1 | 1.3117704 | NM_025130 | hexokinase domain containing 1 |
| Cell cycle |  |  |  |
| CCND2 | 3.5426855 | NM_001759 | cyclin D2 |
| CNTD2 | 2.126494 | NM_024877 | cyclin N-terminal domain containing 2 |
| MDM2 | 1.8906732 | NM_002392 | Mdm2 p53 binding protein homolog (mouse) |
| CDKN1C | 1.6109896 | NM_000076 | cyclin-dependent kinase inhibitor 1C (p57, Kip2) |
| GADD45A | 1.4208784 | NM_001924 | growth arrest and DNA-damage-inducible, alpha |
| YWHAE | 1.3554211 | AK296555 | tyrosine 3-monooxygenase |
| CDK13 | 1.2584047 | NM_031267 | cyclin-dependent kinase 13 |
| ANAPC1 | 1.2567239 | NM_022662 | anaphase promoting complex subunit 1 |
| GADD45G | 1.1424665 | NM_006705 | growth arrest and DNA-damage-inducible, gamma |
| ZFP161 | 1.1084623 | NM_003409 | zinc finger protein 161 homolog (mouse) |
| CDKN2C | -2.4524717 | NM_078626 | cyclin-dependent kinase inhibitor 2C (p18, inhibits CDK4) |
| SFXN2 | -2.4345522 | NM_178858 | sideroflexin 2 |
| CCND3 | -2.4006195 | NM_001760 | cyclin D3 |
| CCNE2 | -2.351235 | NM_057749 | cyclin E2 |
| PLK1 | -2.3180866 | NM_005030 | polo-like kinase 1 |
| ESPL1 | -2.3045168 | NM_012291 | extra spindle pole bodies homolog 1 (S. cerevisiae) |
| E2F2 | -2.2780972 | NM_004091 | E2F transcription factor 2 |
| CCNA2 | -2.0581226 | NM_001237 | cyclin A2 |
| SKP2 | -1.9806805 | NM_032637 | S-phase kinase-associated protein 2 (p45) |
| ORC1 | -1.979352 | NM_004153 | origin recognition complex, subunit 1 |
| CDC25A | -1.933322 | NM_001789 | cell division cycle 25 homolog A (S. pombe) |
| MCM7 | -1.8407898 | NM_005916 | minichromosome maintenance complex component 7 |
| PCNA-AS1 | -1.7084265 | NR_028370 | PCNA antisense RNA 1 (non-protein coding) |
| CDK1 | -1.6983309 | NM_001786 | cyclin-dependent kinase 1 |
| FZR1 | -1.6542406 | NM_016263 | fizzy/cell division cycle 20 related 1 (Drosophila) |
| CCNB1 | -1.6495781 | NM_031966 | cyclin B1 |
| DBF4B | -1.6413393 | NM_145663 | DBF4 homolog B (S. cerevisiae) |
| BUB1B | -1.5819006 | NM_001211 | budding uninhibited by benzimidazoles 1 homolog beta (yeast) |
| HDAC2 | -1.5524826 | NM_001527 | histone deacetylase 2 |
| ORC6 | -1.5479622 | NM_014321 | origin recognition complex, subunit 6 |
| TFDP1 | -1.5272932 | NM_007111 | transcription factor Dp-1 |
| MCM6 | -1.4012833 | NM_005915 | minichromosome maintenance complex component 6 |
| BUB1 | -1.354682 | NM_004336 | budding uninhibited by benzimidazoles 1 homolog (yeast) |
| DBF4 | -1.3184767 | NM_006716 | DBF4 homolog (S. cerevisiae) |
| SMC1A | -1.3124914 | NM_006306 | structural maintenance of chromosomes 1A |
| RBL1 | -1.3119001 | NM_002895 | retinoblastoma-like 1 (p107) |
| PTTG1 | -1.2546062 | NM_004219 | pituitary tumor-transforming 1 |
| RSPO3 | -1.2190771 | NM_032784 | R-spondin 3 |
| CDC7 | -1.2078094 | NM_003503 | cell division cycle 7 homolog (S. cerevisiae) |
| CDK2 | -1.1768713 | NM_001798 | cyclin-dependent kinase 2 |
| CDC25C | -1.1749735 | NM_001790 | cell division cycle 25 homolog C (S. pombe) |
| CCNB2 | -1.1293583 | NM_004701 | cyclin B2 |
| PCNA | -1.0213375 | NM_002592 | proliferating cell nuclear antigen |
| **DNA replication** | | | |
| CCND2 | 3.5426855 | NM_001759 | cyclin D2 |
| CNTD2 | 2.126494 | NM_024877 | cyclin N-terminal domain containing 2 |
| MDM2 | 1.8906732 | NM_002392 | Mdm2 p53 binding protein homolog (mouse) |
| LOC339260 | 1.7083955 | BC041488 | uncharacterized LOC339260 |
| CDKN1C | 1.6109896 | NM_000076 | cyclin-dependent kinase inhibitor 1C (p57, Kip2) |
| GADD45A | 1.4208784 | NM_001924 | growth arrest and DNA-damage-inducible, alpha |
| YWHAE | 1.3554211 | AK296555 | tyrosine 3-monooxygenase |
| CDK13 | 1.2584047 | NM_031267 | cyclin-dependent kinase 13 |
| ANAPC1 | 1.2567239 | NM_022662 | anaphase promoting complex subunit 1 |
| GADD45G | 1.1424665 | NM_006705 | growth arrest and DNA-damage-inducible, gamma |
| ZFP161 | 1.1084623 | NM_003409 | zinc finger protein 161 homolog (mouse) |
| ATAD5 | -1.3161297 | NM_024857 | ATPase family, AAA domain containing 5 |
| BUB1 | -1.354682 | NM_004336 | budding uninhibited by benzimidazoles 1 homolog (yeast) |
| BUB1B | -1.5819006 | NM_001211 | budding uninhibited by benzimidazoles 1 homolog beta (yeast) |
| CCNA2 | -2.0581226 | NM_001237 | cyclin A2 |
| CCNB1 | -1.6495781 | NM_031966 | cyclin B1 |
| CCNB2 | -1.1293583 | NM_004701 | cyclin B2 |
| CCND3 | -2.4006195 | NM_001760 | cyclin D3 |
| CCNE2 | -2.351235 | NM_057749 | cyclin E2 |
| CDC25A | -1.933322 | NM_001789 | cell division cycle 25 homolog A (S. pombe) |
| CDC25C | -1.1749735 | NM_001790 | cell division cycle 25 homolog C (S. pombe) |
| CDC7 | -1.2078094 | NM_003503 | cell division cycle 7 homolog (S. cerevisiae) |
| CDK1 | -2.5203757 | NM_001170406 | cyclin-dependent kinase 1 |
| CDK2 | -1.1768713 | NM_001798 | cyclin-dependent kinase 2 |
| CDKN2C | -2.4524717 | NM_078626 | cyclin-dependent kinase inhibitor 2C (p18, inhibits CDK4) |
| DBF4 | -1.3184767 | NM_006716 | DBF4 homolog (S. cerevisiae) |
| DBF4B | -1.6413393 | NM_145663 | DBF4 homolog B (S. cerevisiae) |
| E2F1 | -2.1694212 | NM_005225 | E2F transcription factor 1 |
| E2F2 | -2.2780972 | NM_004091 | E2F transcription factor 2 |
| ESPL1 | -2.3045168 | NM_012291 | extra spindle pole bodies homolog 1 (S. cerevisiae) |
| FEN1 | -2.0378265 | NM_004111 | flap structure-specific endonuclease 1 |
| FZR1 | -1.6542406 | NM_016263 | fizzy/cell division cycle 20 related 1 (Drosophila) |
| HDAC2 | -1.5524826 | NM_001527 | histone deacetylase 2 |
| LYG2 | -1.3940706 | NM_175735 | lysozyme G-like 2 |
| MCM6 | -1.4012833 | NM_005915 | minichromosome maintenance complex component 6 |
| MCM7 | -1.8407898 | NM_005916 | minichromosome maintenance complex component 7 |
| ORC1 | -1.979352 | NM_004153 | origin recognition complex, subunit 1 |
| ORC6 | -1.5479622 | NM_014321 | origin recognition complex, subunit 6 |
| PCNA | -1.0213375 | NM_002592 | proliferating cell nuclear antigen |
| PCNA-AS1 | -1.7084265 | NR_028370 | PCNA antisense RNA 1 (non-protein coding) |
| PLK1 | -2.3180866 | NM_005030 | polo-like kinase 1 |
| POLA1 | -1.1825066 | NM_016937 | polymerase (DNA directed), alpha 1, catalytic subunit |
| POLA2 | -1.7700844 | NM_002689 | polymerase (DNA directed), alpha 2 (70kD subunit) |
| POLD1 | -1.125701 | NM_002691 | polymerase (DNA directed), delta 1, catalytic subunit 125kDa |
| PRIM1 | -1.1008034 | NM_000946 | primase, DNA, polypeptide 1 (49kDa) |
| PTTG1 | -1.2546062 | NM_004219 | pituitary tumor-transforming 1 |
| RBL1 | -1.3119001 | NM_002895 | retinoblastoma-like 1 (p107) |
| RFC3 | -1.5060596 | NM_181558 | replication factor C (activator 1) 3, 38kDa |
| RFC5 | -1.1276197 | NM_181578 | replication factor C (activator 1) 5, 36.5kDa |
| RNASEH2A | -1.0142603 | NM_006397 | ribonuclease H2, subunit A |
| RNASEH2C | -1.195054 | NM_032193 | ribonuclease H2, subunit C |
| RPA1 | -1.9159093 | NM_002945 | replication protein A1, 70kDa |
| RSPO3 | -1.2190771 | NM_032784 | R-spondin 3 |
| SFXN2 | -2.4345522 | NM_178858 | sideroflexin 2 |
| SKP2 | -1.9806805 | NM_032637 | S-phase kinase-associated protein 2 (p45) |
| SMC1A | -1.3124914 | NM_006306 | structural maintenance of chromosomes 1A |
| TFDP1 | -1.5272932 | NM_007111 | transcription factor Dp-1 |
| **Drug metabolism - other enzymes** | | | |
| XDH | 2.039585 | NM_000379 | xanthine dehydrogenase |
| UPP1 | 1.2207994 | NM_181597 | uridine phosphorylase 1 |
| UGT2B15 | -4.265785 | NM_001076 | UDP glucuronosyltransferase 2 family, polypeptide B15 |
| UGT1A6 | -4.121839 | NM_001072 | UDP glucuronosyltransferase 1 family, polypeptide A6 |
| UGT1A8 | -3.737194 | NM_019076 | UDP glucuronosyltransferase 1 family, polypeptide A8 |
| CES5A | -3.6157665 | NM_145024 | carboxylesterase 5A |
| UGT2B11 | -1.7610216 | NM_001073 | UDP glucuronosyltransferase 2 family, polypeptide B11 |
| CES1 | -1.565978 | NM_001266 | carboxylesterase 1 |
| TK1 | -1.4738722 | NM_003258 | thymidine kinase 1, soluble |
| PRTFDC1 | -1.2683172 | NM_020200 | phosphoribosyl transferase domain containing 1 |
| NAT1 | -1.2251616 | NM_000662 | N-acetyltransferase 1 (arylamine N-acetyltransferase) |
| UGT2B7 | -1.0154257 | NM_001074 | UDP glucuronosyltransferase 2 family, polypeptide B7 |
| **Glycosaminoglycan biosynthesis - chondroitin sulfate** | | | |
| CSGALNACT2 | 2.5119748 | NM_018590 | chondroitin sulfate N-acetylgalactosaminyltransferase 2 |
| XYLT1 | 1.3740206 | NM_022166 | xylosyltransferase I |
| UST | 1.3706522 | NM_005715 | uronyl-2-sulfotransferase |
| CHPF | 1.3238211 |  | chondroitin polymerizing factor |
| WSCD1 | 1.118814 | NM_015253 | WSC domain containing 1 |
| DSEL | 1.0752492 | NM_032160 | dermatan sulfate epimerase-like |
| DSE | 1.0397282 | NM_013352 | dermatan sulfate epimerase |
| CHSY3 | -2.3888593 | NM_175856 | chondroitin sulfate synthase 3 |
| **Metabolism of xenobiotics by cytochrome P450** | | |  |
| GDAP1L1 | 2.924951 | NM_024034 | ganglioside-induced differentiation-associated protein 1-like 1 |
| CYP1A1 | 1.99156 | NM_000499 | cytochrome P450, family 1, subfamily A, polypeptide 1 |
| DHDH | 1.4982519 | NM_014475 | dihydrodiol dehydrogenase (dimeric) |
| UGT2B15 | -4.265785 | NM_001076 | UDP glucuronosyltransferase 2 family, polypeptide B15 |
| UGT1A6 | -4.121839 | NM_001072 | UDP glucuronosyltransferase 1 family, polypeptide A6 |
| GSTA5 | -3.865056 | NM_153699 | glutathione S-transferase alpha 5 |
| UGT1A8 | -3.737194 | NM_019076 | UDP glucuronosyltransferase 1 family, polypeptide A8 |
| UGT2B11 | -1.7610216 | NM_001073 | UDP glucuronosyltransferase 2 family, polypeptide B11 |
| ALDH3B2 | -1.5447574 | NM_000695 | aldehyde dehydrogenase 3 family, member B2 |
| EPHX1 | -1.4821224 | NM_000120 | epoxide hydrolase 1, microsomal (xenobiotic) |
| GDAP1 | -1.1703978 | NM_018972 | ganglioside-induced differentiation-associated protein 1 |
| ALDH3A1 | -1.0939369 | NM_000691 | aldehyde dehydrogenase 3 family, member A1 |
| UGT2B7 | -1.0154257 | NM_001074 | UDP glucuronosyltransferase 2 family, polypeptide B7 |
| GSTM2P1 | -1.0133467 | NR_002932 | glutathione S-transferase mu 2 (muscle) pseudogene 1 |
| **Nitrogen metabolism** | |  |  |
| CA13 | 4.214135 | NM_198584 | carbonic anhydrase XIII |
| PTX3 | 2.3451385 | NM_002852 | pentraxin 3, long |
| FLJ43315 | 1.8176241 | NR_033856 | asparagine synthetase pseudogene |
| LOC442028 | 1.5556688 | NR_037597 | uncharacterized LOC442028 |
| AMT | 1.5058956 | NM_000481 | aminomethyltransferase |
| CTH | 1.380784 | NM_001902 | cystathionase (cystathionine gamma-lyase) |
| GLS | 1.0751734 | AF097492 | glutaminase |
| AMTN | -3.6812277 | NM_212557 | amelotin |
| CA11 | -1.0331144 | NM_001217 | carbonic anhydrase XI |
| **One carbon pool by folate** | |  |  |
| MTHFR | 1.7510767 | NM_005957 | methylenetetrahydrofolate reductase (NAD(P)H) |
| AMT | 1.5058956 | NM_000481 | aminomethyltransferase |
| ALDH1L2 | 1.0016279 | NM_001034173 | aldehyde dehydrogenase 1 family, member L2 |
| AMTN | -3.6812277 | NM_212557 | amelotin |
| DHFR | -1.7413721 | NM_000791 | dihydrofolate reductase |
| MTR | -1.2951188 | NM_000254 | 5-methyltetrahydrofolate-homocysteine methyltransferase |
| MTRNR2L1 | -1.2231417 | NM_001190452 | MT-RNR2-like 1 |
| LOC541467 | -1.2144728 | BC045815 | uncharacterized LOC541467 |
| MTRNR2L7 | -1.186749 | NM_001190489 | MT-RNR2-like 7 |
| TYMS | -1.0349674 | NM_001071 | thymidylate synthetase |
| **Pancreatic secretion** | | | |
| ATP2B4 | 3.182561 | NM_001001396 | ATPase, Ca++ transporting, plasma membrane 4 |
| PRSS2 | 2.6647987 | NM_002770 | protease, serine, 2 (trypsin 2) |
| ICA1 | 2.1959887 | NM_004968 | islet cell autoantigen 1, 69kDa |
| CCK | 2.0106225 | NM_000729 | cholecystokinin |
| BAIAP3 | 1.9779124 | NM_003933 | BAI1-associated protein 3 |
| PRSS3 | 1.9748898 | NM_002771 | protease, serine, 3 |
| ICA1 | 1.9650488 | NM_004968 | islet cell autoantigen 1, 69kDa |
| PRSS22 | 1.8248572 | NM_022119 | protease, serine, 22 |
| MCTP1 | 1.7965755 | NM_024717 | multiple C2 domains, transmembrane 1 |
| LOC100128402 | 1.7920489 | AK124574 | uncharacterized LOC100128402 |
| ATP1B2 | 1.7542062 | NM_001678 | ATPase, Na+/K+ transporting, beta 2 polypeptide |
| KCNQ1 | 1.6659322 | NM_000218 | potassium voltage-gated channel, KQT-like subfamily, member 1 |
| ATP2B2 | 1.5540252 | NM_001001331 | ATPase, Ca++ transporting, plasma membrane 2 |
| ATP2A1 | 1.5025773 | NM_173201 | ATPase, Ca++ transporting, cardiac muscle, fast twitch 1 |
| PLA2G4C | 1.4468822 | NM_003706 | phospholipase A2, group IVC (cytosolic, calcium-independent) |
| SCT | 1.4244256 | NM_021920 | secretin |
| CELA3A | 1.278377 | NM_005747 | chymotrypsin-like elastase family, member 3A |
| SLC9A1 | 1.1486764 | BC012121 | solute carrier family 9 (sodium/hydrogen exchanger), member 1 |
| KCNQ1OT1 | 1.09126 | NR_002728 | KCNQ1 opposite strand/antisense transcript 1 (non-protein coding) |
| PLA2G2F | 1.0774126 | NM_022819 | phospholipase A2, group IIF |
| ITPR2 | 1.0662332 | NM_002223 | inositol 1,4,5-trisphosphate receptor, type 2 |
| KCNQ1 | -5.2146387 | AF003743 | potassium voltage-gated channel, KQT-like subfamily, member 1 |
| SLC9A1 | -4.8376637 |  | solute carrier family 9 (sodium/hydrogen exchanger), member 1 |
| ICA1L | -4.312938 | NM_178231 | islet cell autoantigen 1,69kDa-like |
| ATP2B3 | -4.1298575 | NM_001001344 | ATPase, Ca++ transporting, plasma membrane 3 |
| SLC9A11 | -4.0828147 | NM_178527 | solute carrier family 9, member 11 |
| FLJ43879 | -3.6268191 | NR_034162 | FLJ43879 protein |
| PRSS35 | -3.001779 | NM_153362 | protease, serine, 35 |
| ATP2B2 | -2.568235 | NM_001001331 | ATPase, Ca++ transporting, plasma membrane 2 |
| LOC100292680 | -1.936409 | NR_028415 | uncharacterized LOC100292680 |
| FXYD2 | -1.7904873 | NM_021603 | FXYD domain containing ion transport regulator 2 |
| RAB3D | -1.4411807 | NM_004283 | RAB3D, member RAS oncogene family |
| SLC12A2 | -1.3794622 | NM_001046 | solute carrier family 12 |
| PLCB4 | -1.375308 | NM_001172646 | phospholipase C, beta 4 |
| BST1 | -1.2988052 | NM_004334 | bone marrow stromal cell antigen 1 |
| PLA2G1B | -1.2563686 | NM_000928 | phospholipase A2, group IB (pancreas) |
| LOC100507437 | -1.2549076 | XR_110258 | uncharacterized LOC100507437 |
| ADCY6 | -1.1729965 | NM_015270 | adenylate cyclase 6 |
| SLC4A4 | -1.1353769 | NM_003759 | solute carrier family 4, sodium bicarbonate cotransporter |
| SLC12A8 | -1.0671072 | NM_024628 | solute carrier family 12 (potassium/chloride transporters) |
| KCNMA1 | -1.0334101 | NM_001014797 | potassium large conductance calcium-activated channel |
| **Pentose and glucuronate interconversions** | | |  |
| CAMTA1 | 2.5883403 | NM_015215 | calmodulin binding transcription activator 1 |
| DHDH | 1.4982519 | NM_014475 | dihydrodiol dehydrogenase (dimeric) |
| ALDH8A1 | 1.4613128 | NM_022568 | aldehyde dehydrogenase 8 family, member A1 |
| UGT2B15 | -4.265785 | NM_001076 | UDP glucuronosyltransferase 2 family, polypeptide B15 |
| UGT1A6 | -4.121839 | NM_001072 | UDP glucuronosyltransferase 1 family, polypeptide A6 |
| UGT1A8 | -3.737194 | NM_019076 | UDP glucuronosyltransferase 1 family, polypeptide A8 |
| UGT2B11 | -1.7610216 | NM_001073 | UDP glucuronosyltransferase 2 family, polypeptide B11 |
| UGT2B7 | -1.0154257 | NM_001074 | UDP glucuronosyltransferase 2 family, polypeptide B7 |
| **Starch and sucrose metabolism** | |  |  |
| SNAP91 | 4.384363 | NM_014841 | synaptosomal-associated protein, 91kDa homolog (mouse) |
| HK2 | 3.4536276 | NM_000189 | hexokinase 2 |
| AMY1C | 1.6465478 | NM_001008219 | amylase, alpha 1C (salivary) |
| PICALM | 1.3348236 | NM_007166 | phosphatidylinositol binding clathrin assembly protein |
| HKDC1 | 1.3117704 | NM_025130 | hexokinase domain containing 1 |
| UGT2B15 | -4.265785 | NM_001076 | UDP glucuronosyltransferase 2 family, polypeptide B15 |
| UGT1A6 | -4.121839 | NM_001072 | UDP glucuronosyltransferase 1 family, polypeptide A6 |
| UGT1A8 | -3.737194 | NM_019076 | UDP glucuronosyltransferase 1 family, polypeptide A8 |
| UGT2B11 | -1.7610216 | NM_001073 | UDP glucuronosyltransferase 2 family, polypeptide B11 |
| PGAP3 | -1.1818914 | NM_033419 | post-GPI attachment to proteins 3 |
| ENPP1 | -1.1542635 | NM_006208 | ectonucleotide pyrophosphatase/phosphodiesterase 1 |
| LCTL | -1.1055498 | NM_207338 | lactase-like |
| UGT2B7 | -1.0154257 | NM_001074 | UDP glucuronosyltransferase 2 family, polypeptide B7 |
| **Synthesis and degradation of ketone bodies** | | | |
| HMGCLL1 | -2.1726875 | NM_019036 | 3-hydroxymethyl-3-methylglutaryl-CoA lyase-like 1 |
| BDH1 | -1.5011206 | NM_203314 | 3-hydroxybutyrate dehydrogenase, type 1 |
| BDH2 | -1.2657132 | NM_020139 | 3-hydroxybutyrate dehydrogenase, type 2 |
| **Tropane, piperidine and pyridine alkaloid biosynthesis** | | | |
| AOC3 | 2.7352333 | NM_003734 | amine oxidase, copper containing 3 (vascular adhesion protein 1) |
| AOC2 | 2.3255157 | NM_001158 | amine oxidase, copper containing 2 (retina-specific) |
| CES5A | -3.6157665 | NM_145024 | carboxylesterase 5A |
| CES1 | -1.565978 | NM_001266 | carboxylesterase 1 |
| PLD5 | -1.5234842 | NM_152666 | phospholipase D family, member 5 |
| CES1 | -1.3035107 | NM_001025195 | carboxylesterase 1 |
| PLD5 | -1.1043105 | NM_152666 | phospholipase D family, member 5 |

All these pathways: P<0.05
